# Phosphorylation of Def Regulates Nucleolar p53 Turnover and Cell Cycle Progression through Def Recruitment of Calpain3

**DOI:** 10.1371/journal.pbio.1002555

**Published:** 2016-09-22

**Authors:** Yihong Guan, Delai Huang, Feng Chen, Ce Gao, Ting Tao, Hui Shi, Shuyi Zhao, Zuyuan Liao, Li Jan Lo, Yingchun Wang, Jun Chen, Jinrong Peng

**Affiliations:** 1 MOE Key Laboratory for Molecular Animal Nutrition, College of Animal Sciences, Zhejiang University, Hangzhou, China; 2 State Key Laboratory of Molecular Developmental Biology, Institute of Genetics and Developmental Biology, Chinese Academy of Sciences, China; 3 College of Life Sciences, Zhejiang University, Hangzhou, China; St. Jude Children's Research Hospital, UNITED STATES

## Abstract

Digestive organ expansion factor (Def) is a nucleolar protein that plays dual functions: it serves as a component of the ribosomal small subunit processome for the biogenesis of ribosomes and also mediates p53 degradation through the cysteine proteinase calpain-3 (CAPN3). However, nothing is known about the exact relationship between Def and CAPN3 or the regulation of the Def function. In this report, we show that CAPN3 degrades p53 and its mutant proteins p53^A138V^, p53^M237I^, p53^R248W^, and p53^R273P^ but not the p53^R175H^ mutant protein. Importantly, we show that Def directly interacts with CAPN3 in the nucleoli and determines the nucleolar localisation of CAPN3, which is a prerequisite for the degradation of p53 in the nucleolus. Furthermore, we find that Def is modified by phosphorylation at five serine residues: S50, S58, S62, S87, and S92. We further show that simultaneous phosphorylations at S87 and S92 facilitate the nucleolar localisation of Capn3 that is not only essential for the degradation of p53 but is also important for regulating cell cycle progression. Hence, we propose that the Def-CAPN3 pathway serves as a nucleolar checkpoint for cell proliferation by selective inactivation of cell cycle-related substrates during organogenesis.

## Abstract 2

During organogenesis, cell proliferation is controlled not only by many known regulatory networks but also by the newly identified Def-CAPN3 nucleolar protein degradation pathway.

## Author Summary

The nucleolus is primarily known as the subcellular organelle for the biogenesis of the ribosomal small and large subunits in eukaryotic cells. However, it is also increasingly evident that many nucleolar proteins also play essential roles in other biological processes. Here, we show that Digestive organ expansion factor (Def), a nucleolar protein, determines the nucleolar localisation of the cysteine proteinase CAPN3, and they form a complex that degrades the tumor suppressor protein p53, an essential regulator of the cell cycle, in the nucleolus. Furthermore, we show that Def is modified by phosphorylation at five serine residues and that Def phosphorylation modification facilitates the nucleolar localization of Capn3, which is also important for regulating cell cycle progression. Hence, we propose that the Def-CAPN3 pathway serves as a nucleolar checkpoint of cell proliferation by selective inactivation of cell cycle-related substrates during organogenesis, revealing a novel biological role for the nucleolus.

## Introduction

The nucleolus is a subcellular organelle primarily known for the biogenesis of the ribosomal small and large subunits in eukaryotic cells [1,2]. However, bioinformatic analyses of nucleolar proteomic studies in cultured human HeLa cells [3,4], *Arabidopsis* [5], and human T cells [6] have shown that a mere 30% of nucleolar proteins (approximately 200 from Arabidopsis and approximately 700 from human cells) have functions directly related to the production of ribosomal subunits; the rest are involved in a variety of biochemical processes including cell cycle control, stress response, and the biogenesis of ribonucleoproteins. It is increasingly evident that many nucleolar proteins have dual functions. For example, nucleophosmin functions not only in ribosome biogenesis but also in duplication of centrosomes [7] and stress response [8]. Nucleolin is believed to regulate every step of ribosome biosynthesis and is also known to be a multifunctional protein that affects mRNA stability and translation [9]. In addition to its role in rRNA maturation, the DEAD-box RNA helicase DDX21 promotes the expression of snoRNAs and other ribosomal proteins [10]. However, the exact biological functions of many nucleolar proteins remain elusive.

Digestive organ expansion factor (Def) is a nucleolar protein that is conserved across species including yeast, *Drosophila*, *Arabidopsis*, zebrafish, mice, and humans [11–14]. In zebrafish, the loss of function of Def (*def*^*-/-*^) causes digestive organ hypoplasia due to cell cycle arrest but not due to apoptosis [11]. This outcome is in part attributed to the stabilisation of the p53 protein in *def*^*-/-*^ that in turn directly transactivates the expression of the p53 isoform Δ113p53, the function of which is to antagonise p53 apoptotic activity [15–18]. Strikingly, the stabilised p53 in *def*^*-/-*^ accumulates in the nucleolus [17]. This is explained by the finding that Def cooperates with the cysteine protease calpain3 (CAPN3) to degrade p53 [17]. Further investigations showed that Def haploinsufficiency in *def*^+/-^ heterozygous fish activates the p53-dependent transforming growth factor β signalling pathway and leads to scar formation after partial hepatectomy [19]. In addition to its role in the regulation of p53 function, Def is also involved in pre-rRNA processing in different organisms [12–14,20]. However, the precise molecular mechanism of Def’s regulation of p53 stability, cell cycle progression, and small subunit biogenesis remains unclear. In this study, we investigated three lines of inquiry: (i) Is p53 a direct target of CAPN3? (ii) What is the relationship between Def and CAPN3? (iii) How is Def function regulated? In essence, we showed that Def determines the nucleolar localisation of CAPN3/Capn3b, allowing it to achieve its nucleolar function. Def protein is phosphorylated at multiple serine residues, and these modifications work in different combinations to modulate the function of the Def-CAPN3 complex on liver development, p53 degradation, and cell cycle progression. We also discuss the importance of the nucleolar Def-CAPN3 protein degradation pathway in the regulation of cell cycle progression during organogenesis.

## Results

### Wild-Type p53, but Not Mutant p53^R175H^, Is a Substrate of CAPN3

**Fig 1:**
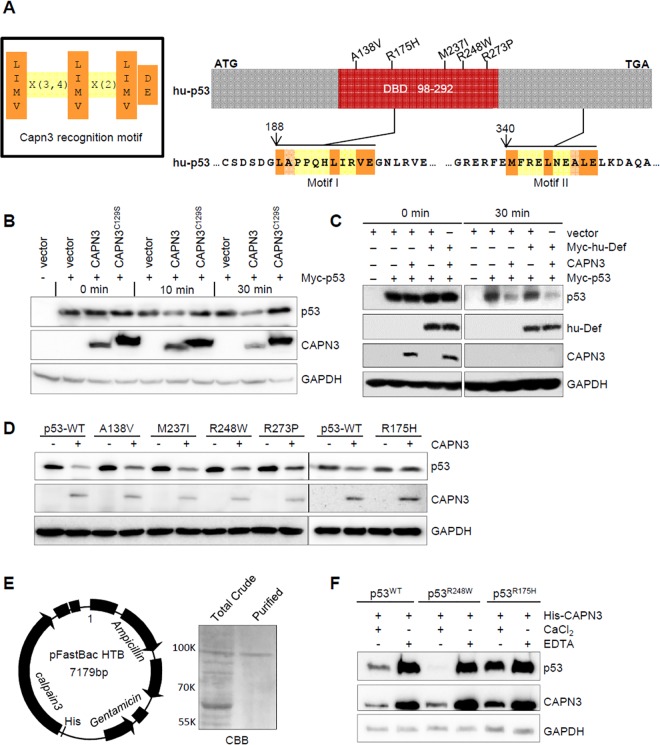
Wild-type p53, but not the mutant p53^R175H^, is a substrate of CAPN3. **(A)** Left panel: the CAPN3 recognition motif. Right panel: upper panel shows the simplified hu-p53 protein with five frequent mutated sites in the DNA-binding domain (DBD) highlighted. Lower panels depict the two putative CAPN3 recognition regions in p53. Numbers denote the position of amino acids in p53. ATG, start codon; TGA, stop codon. **(B–D)** In vitro assay of p53 degradation by CAPN3 or CAPN3^C129S^. CAPN3, CAPN3^C129S^, Myc-hu-Def, Myc-p53, and its mutant derivatives were expressed in 293T cells, respectively. Protein extracts were mixed as indicated and the mixture was incubated in the reaction buffer containing 5 mM CaCl_2_ at 37°C for 0, 10, 30 min **(B)**, or 30 min **(C),** or 45 min **(D)**. GAPDH: loading control. Immunodetection of p53 was achieved by using a combination of monoclonal antibodies PAb240 and PAb1620 **(B, D)**, or Anti-Myc tag antibody 9E10 **(C)**. GAPDH was recognised by mouse monoclonal antibody 5-E10 **(B)** or rabbit monoclonal antibody EPR1977Y **(C, D)**. **(E)** Left panel: diagram showing the CAPN3-pFastBac plasmid (baculovirus expression system) for transfecting the SF9 cells to express His-CAPN3. Right panel: Coomassie brilliant blue (CBB) staining picture showing total protein crude extract (total crude) and purified His-CAPN3 using the Ni-NTA agarose beads (purified). **(F)** In vitro assay of the degradation of Myc-p53^WT^-HA, Myc-p53^R248W^-HA, or Myc-p53^R175H^-HA by the purified His-CAPN3 in the presence of 10 mM CaCl_2_ or 20 mM EDTA as indicated at 37°C for 45 min. GAPDH: loading control. p53 was detected by a combination of monoclonal antibodies PAb240 and Pab1620. GAPDH was recognised by mouse monoclonal antibody 5-E10.

p53 has been shown to be a substrate of conventional cytoplasmic calpains such as CAPN2 [21–23]. We have previously shown that knockdown of CAPN3 in human cells or Capn3b in zebrafish elevates the level of p53 protein [17]. Bioinformatic analysis based on the pattern [L/I/M/V]X(3 or 4)[L/I/M/V/]X(2)[L/I/M/V]D/E] (Fig 1A, left panel) [24] identified two putative CAPN3 recognition motifs in p53 (Fig 1A, right panel and S1A Fig). To determine whether the p53 protein is a direct substrate of CAPN3, we transfected 293T cells with plasmids expressing Myc-p53, wild-type (WT) CAPN3, and bio-inactive CAPN3^C129S^, respectively, and extracted the total proteins. We mixed the total proteins containing over-expressed Myc-p53 with those containing CAPN3 or CAPN3^C129S^ and incubated the mixture at 37°C for 0, 10, or 30 min. Western blot analysis showed that CAPN3—but not CAPN3^C129S^—caused a decrease in the level of p53 protein (Fig 1B). We also examined the effect of human Def (hu-Def) on CAPN3 activity and found that the CAPN3 activity on p53 in this in vitro assay system was Def-independent (Fig 1C).

p53 is frequently mutated in tumour cells, and different p53 mutants show different susceptibilities to conventional calpains [22]. To determine the susceptibility of mutant p53 to CAPN3, we incubated the protein extracts that contained CAPN3 with different p53 mutant proteins such as A138V, R175H, M237I, R248W, and R273P extracted from the corresponding plasmid-transfected 293T cells. Western blot analysis showed that, except for p53^R175H^, CAPN3 efficiently cleaved p53, p53^A138V^, p53^M237I^, p53^R248W^, and p53^R273P^ (Fig 1D). Next, we cloned CAPN3 into pFastBac vector in-frame to the 6xHis-tag (Fig 1E, left panel) and transfected the plasmid into SF9 eukaryotic expression cell system to cause the expression of His-CAPN3. The expressed His-CAPN3 was purified with Ni-NTA agarose beads by eluting with 500 mM of imidazole solution (Fig 1E, right panel and S1B Fig). The purified His-CAPN3 was incubated with p53, p53^R248W^, and p53^R175H^. Clearly, CAPN3 degraded p53 and p53^R248W^, but not p53^R175H^ (Fig 1F), which is consistent with our previous report that p53^R143H^, the human p53^R175H^ counterpart in zebrafish, is resistant to Def-mediated degradation [17]. Furthermore, we found that CAPN3 activity on p53 and p53^R248W^ was calcium-dependent (Fig 1F).

### Def Directly Interacts with CAPN3 to Form a Complex in the Nucleolus

**Fig 2:**
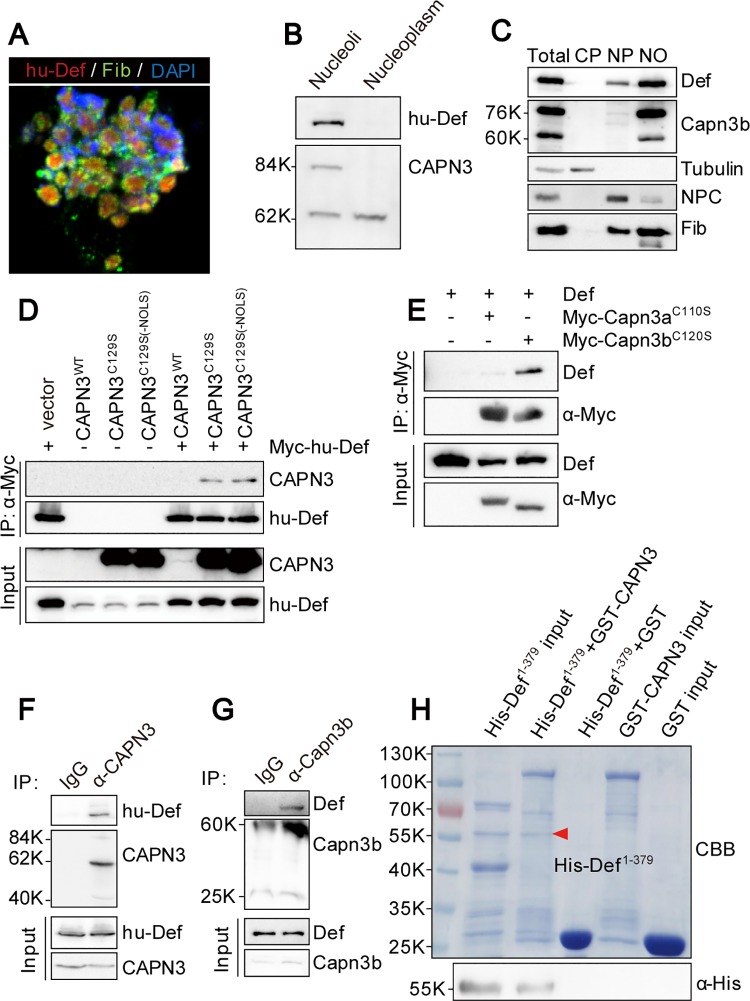
Def directly interacts with CAPN3, and they form a complex in the nucleolus. **(A)** Co-immunostaining of the endogenous hu-Def (red), Fib (green), and DAPI (blue) in the isolated nucleoli from the cultured HepG2 cells. **(B)** Western blot of the endogenous hu-Def and CAPN3 in the nucleoplasmic and nucleolar fractions from HepG2 cells. **(C)** Western blot of the endogenous Def, Capn3b, Tubulin, NPC, and Fib in different fractions as indicated isolated from the adult wild-type zebrafish liver. CP, cytoplasm; NP, nucleoplasm; NO, nucleoli; NPC, nuclear pore complex. **(D)** Co-IP of Myc-hu-Def and CAPN3. 293T cells were co-transfected with *Myc*-*hu-Def* and wild-type *CAPN3* or mutant *CAPN3* (*CAPN3*^*C129S*^ and *CAPN3*^*C129S-ΔNOLS*^) plasmids. Total protein was extracted at 72 h and incubated with Myc-beads. Antibodies against CAPN3 and hu-Def were used in western blotting. **(E)** Co-IP of zebrafish Def and Myc-Capn3a^C110S^ or Myc-Capn3b^C120S^. 293T cells were co-transfected with zebrafish *def* and *Myc-Capn3a*^*C110S*^ or *Myc-Capn3b*^*C120S*^ plasmids, respectively. Antibodies against zebrafish Def and the Myc-tag were used in western blotting. **(F)** Co-IP of the endogenous hu-Def and CAPN3 in HepG2 cells. Total protein extract was incubated with protein A/G agarose beads conjugated with anti-CAPN3 goat polyclonal antibody (COP-080049). CAPN3 was detected by a rabbit polyclonal antibody (No. 38963). **(G)** Co-IP of the endogenous Def and Capn3b in the isolated adult zebrafish liver nulceoli. Nucleolar protein extract was incubated with protein A/G agarose beads conjugated with anti-Capn3b antibody. **(H)** Def directly interacts with CAPN3. GST-pulldown was performed by incubating the purified His-Def^1-379^ with the purified GST, or GST-tagged wild-type CAPN3 immobilised on GST resin. Eluted proteins were stained either with Coomassie blue (CBB) (upper panel) or western blotting using an antibody against the His-tag (lower panel).

We have shown previously that Def is a nucleolar protein and mediates p53 degradation through CAPN3 in human and Capn3b in zebrafish [17]; however, the subcellular localisation of CAPN3/Capn3b is not clear. We isolated nucleoli from cultured HepG2 cells and confirmed that the nucleoli contained both hu-Def and the nucleolar marker Fibrillarin (Fib) (Fig 2A). Results of western blot analysis performed to compare the distribution of hu-Def and CAPN3 in the nucleoli and nucleoplasm showed that hu-Def was clearly enriched in the nucleoli. Interestingly, two CAPN3 isoforms were detected in the nucleoli extract, with one ~84 kD, the bioactive form [25], detected mainly in the nucleoli and the other ~62 kD in both fractions (Fig 2B). In the zebrafish genome, there are two *capn3* homolog genes: *capn3a* and *capn3b*. We previously showed that Capn3b—but not Capn3a—teams with Def to mediate p53 degradation [17]. We examined the expression of both Capn3a and Capn3b but found Capn3a to be undetectable in the adult liver, whereas Capn3b was expressed as two variants (76 kD and 60 kD) that were detected as Fib mainly in the nucleoli fraction and barely detectable in the nucleoplasm fraction (Fig 2C). As controls, the nuclear pore complex was detected mainly in the nucleoplasm fraction, and tubulin was detected mainly in the cytoplasmic fraction (Fig 2C).

Upon overexpression in 293T cells, WT CAPN3 protein was extremely unstable due to its rapid autolysis (Fig 2D) [26]. To circumvent this, we generated the bio-inactive but more stable mutant CAPN3^C129S^ for our co-immunoprecipitation (Co-IP) experiment. The result showed that hu-Def strongly interacted with mutant CAPN3^C129S^ (Fig 2D), suggesting that enzymatic activity is not necessary for Def-CAPN3 interaction. CAPN3 harbours a putative nucleolar localisation motif KKKTKP (NOLS) [17]. We found that deletion of this motif (the CAPN3^C129S-ΔNOLS^ mutant) did not affect the interaction between hu-Def and CAPN3 (Fig 2D). We also generated mutants corresponding to zebrafish Capn3a (Capn3a^C110S^) and Capn3b (Capn3b^C120S^) and over-expressed them in 293T cells. The Co-IP result showed that Capn3b—but not Capn3a—strongly interacted with zebrafish Def (Fig 2E). To determine whether the endogenous hu-Def and CAPN3 form a complex in the nucleolus, we performed a Co-IP experiment on total protein from cultured HepaG2 cells using an antibody against CAPN3. Western blot analysis showed that the endogenous hu-Def was co-precipitated with CAPN3 (Fig 2F). We also isolated nucleoli from the adult zebrafish liver and extracted nucleolar protein for Co-IP and found that Def was co-precipitated with Capn3b (Fig 2G).

To determine whether hu-Def directly interacts with CAPN3, we expressed His-tagged Def^1-379^ and GST-tagged full-length CAPN3 in *Escherichia coli* and purified His- Def^1-379^ with Ni-NTA agarose beads and GST-CAPN3 with GST beads. We then mixed the purified His-Def^1-379^ with the GST beads bound with GST or GST-CAPN3 followed by elution with SDS lysis buffer. Both Coomassie brilliant blue staining (Fig 2H, upper panel) and western blot analysis (Fig 2H, lower panel) showed that GST-CAPN3 protein—but not GST protein—retained His- Def^1-379^ on the beads.

### Def Determines CAPN3 Nucleolar Localisation

CAPN3 harbours a putative nucleolar localisation signal (NOLS). Having shown that this motif is dispensable for CAPN3-Def interaction (Fig 2D), we then analysed the protein in the nucleoli isolated from cells transfected with the *CAPN3*^*C129S-ΔNOLS*^ plasmid and found that deletion of the NOLS motif did not affect the nucleolar localisation of CAPN3 (S2A Fig). This suggests that this NOLS is not essential or is not the sole determinant of nucleolar localisation of CAPN3.

**Fig 3:**
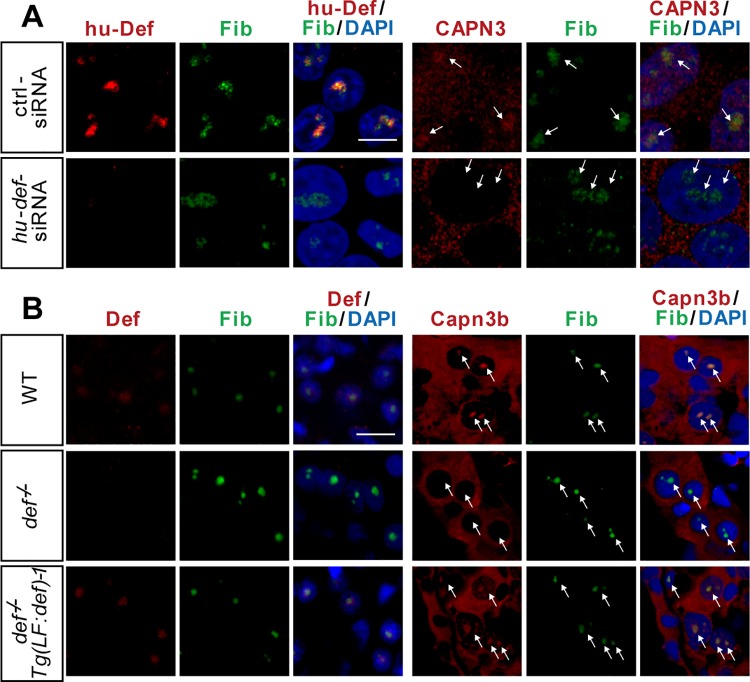
Def determines the nucleolar localisation of Capn3. **(A)** Co-immunostaining of the endogenous hu-Def and CAPN3 in 293T cells. Cells were transfected with control siRNA or siRNAs specifically targeting *hu-def* and cultured for 24 h. **(B)** Co-immunostaining of the endogenous Def and Capn3b in the liver of 6.5-dpf WT, *def*^*-/-*^, and *def*^*-/-*^ *Tg(fabp10a*:*def)-1* (labelled as *Tg(LF*:*def)-1*) zebrafish.

Next, we sought to determine whether formation of the Def-CAPN3 complex is a prerequisite for the nucleolar localisation of CAPN3. We knocked down the expression of hu-Def in 293T cells by *hu-def*–specific siRNA (Fig 3A and S2B Fig) [17] and found that CAPN3 was restricted to the cytoplasm (Fig 3A). We then analysed the subcellular localisation of Capn3b in the zebrafish *def*^*-/-*^ mutant and found that the nucleoli of the mutant liver cells lacked the Capn3b signal (Fig 3B, S1 Table). We have previously shown that *Tg(fabp10a*:*def)* rescues the small liver phenotype conferred by *def*^*-/-*^ [20]. We thus analysed the subcellular localisation of Capn3b in the *def*^*-/-*^ *Tg(fabp10a*:*def)* fish at 6.5 d post fertilisation (dpf) by co-immunostaining Fib and Capn3b. We found that the nucleolar localisation of Capn3b was restored in the hepatocytes of *def*^*-/-*^ *Tg(fabp10a*:*def)* (Fig 3B, S1 Table). We concluded that the translocation of CAPN3/Capn3b into the nucleolus is Def-dependent.

### Def N-terminus Is Modified by Phosphorylation

**Fig 4:**
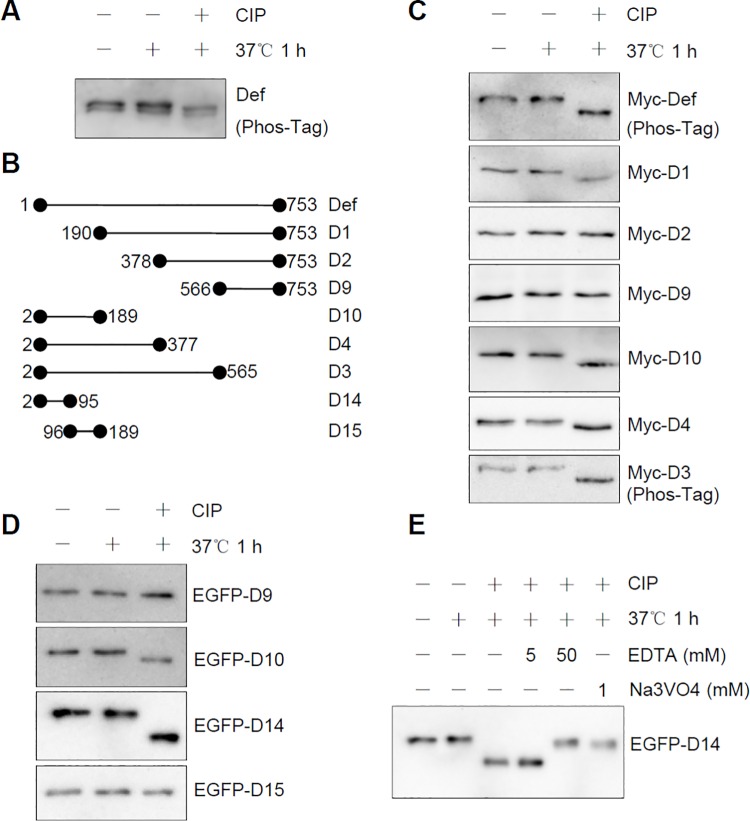
Def is phosphorylated at its N-terminus. **(A)** Western blot of endogenous Def in protein extracts from 32-hpf embryos treated with (+) or without (-) CIP for 1 h at 37°C. **(B)** Diagram showing different Def derivatives used in **(C, D)**. **(C and D)** Western blot to detect Myc-tagged (using anti-Myc antibody) **(C)** or EGFP-tagged (using anti-EGFP antibody). **(D)** Def derivatives treated with (+) or without (-) CIP. **(E)** EGFP-D14 was mixed with EDTA or Na_3_VO_4_ prior to addition of CIP. An anti-EGFP antibody was used in western blot. Phos-Tag: Phos-Tag gel for western blot **(A, C)**.

We reported previously that Def is not modified by glycosylation or ubiquitination/sumoylation [27]. To determine whether endogenous Def, like many other nucleolar proteins such as MPP10 [28], is a phosphorylated protein, we extracted the total protein from embryos at 32 h post fertilisation (hpf) and treated the samples with calf intestinal alkaline phosphatase (CIP). Western blot analysis showed that endogenous Def displayed two bands that exhibited faster migration upon CIP treatment (Fig 4A), thus showing that Def is a phosphorylated protein. The double bands might indicate that endogenous Def exists in two forms or that a portion of endogenous Def is modified by another uncharacterised chemical modification that is resistant to CIP treatment. Def in zebrafish is composed of 753 amino acid (AA) residues. To determine which region of Def is phosphorylated, we expressed different Def-derivatives with N-terminus or C-terminus truncations (Fig 4B). Western blot analysis revealed that D3 (2–565 AA), D4 (2–377 AA), D10 (2–189 AA), and D1 (190–753 AA)—but not D2 (378–753 AA) and D9 (566–753)—had faster migration that was CIP-dependent (Fig 4C). By cross-comparison, we concluded that the regions between 1-189AA (represented by D10) and 190-377AA are modified by phosphorylation. To determine which region in D10 was phosphorylated, we divided D10 (encoding 188 AA) into D14 (2–95 AA) and D15 (96–189 AA) (Fig 4B) and fused them to enhanced green fluorescent protein (EGFP). We treated EGFP-D14 and EGFP-D15 with CIP. Western blot analysis showed that EGFP-D14—but not EGFP-D15—was targeted for phosphorylation (Fig 4D). The effect of CIP treatment on EGFP-D14 mobility could be abrogated by the addition of 50 mM of ethylene diaminetetraacetic acid (EDTA) (an inhibitor of CIP), but not by 5 mM EDTA (Fig 4E). Treatment with 1 mM of sodium orthovanadate (Na_3_VO_4_, another inhibitor of CIP) also blocked the CIP treatment effect on EGFP-D14 mobility (Fig 4E). These data suggest that Def is phosphorylated between 2–95 AA of the protein.

### S50, S58, S62, S87, and S92 Are Phosphorylation Sites in Def

**Fig 5:**
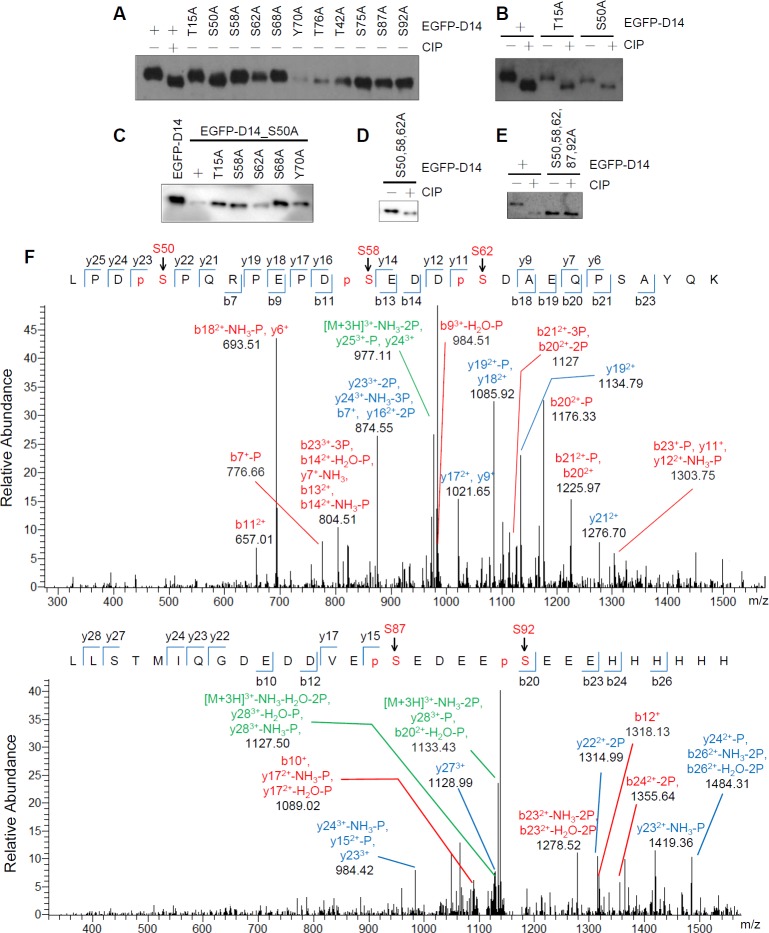
S50, S58, S62, S87, and S92 are modified by phosphorylation. **(A–E)** Western blot (using an anti-EGFP antibody) of EGFP-D14 and its various single mutant **(A, B)**, or EGFP-D14 and various double mutant protein derived from EGFP-D14_S50A **(C)**, or EGFP-D14_S50,58,62A triple mutant protein **(D)**, or EGFP-D14_S50,58,62,87,92A penta mutant protein **(E)** to analyse the gel mobility shift of these EGFP-D14 derivatives treated with (+) or without (-) CIP as shown. **(F)** Mass spectrum analysis of the EGFP-D14 peptide expressed by 293T cells. S50, S58, and S62 (upper panel), S87 and S92 (lower panel) five serine residues (pS) were identified to be modified by phosphorylation.

As shown above, Def phosphorylation occurs in its N-terminal end as defined by D14. Because phosphorylation modification often happens on serine (S), threonine (T), and tyrosine (Y) residues, we generated alanine (A) substitution mutant constructs corresponding to each of these three residues types (a total of seven serine, three threonine, and one tyrosine residue in D14) in EGFP-D14. Western blot analysis showed an obvious band shift for the S50A (i.e., Ser^50^ to Ala^50^) mutant protein (Fig 5A), suggesting that S50 is a phosphorylation site in Def. We next treated both EGFP-D14_T15A (as a control) and EGFP-D14_S50A with CIP and found that CIP-treated EGFP-D14_S50A migrated faster than untreated EGFP-D14_S50A, suggesting that this peptide has additional phosphorylation site(s) (Fig 5B). We carried out alanine substitutions in the background of EGFP-D14_S50A and found that both the S58A (i.e., EGFP-D14_S50,58A double mutant) and S62A (i.e., EGFP-D14_S50,62A double mutant) mutations resulted in subtle but clear gel mobility shifts (Fig 5C). We then constructed the EGFP-D14_S50,58,62A triple mutant and were surprised to find that this mutant protein continued to display a clear shift upon CIP-treatment (Fig 5D). We made further mutations in the triple mutant background and finally found that the EGFP-D14_S50,58,62,87,92A mutant protein did not show a further shift upon treatment with CIP (Fig 5E).

To determine the location of Def phosphorylation sites unequivocally, we transfected 293T cells with the plasmid *pCS2*^*+*^*-((6×His)-EGFP-D14-(6×His))* (6×His tag fused to EGFP-D14 at both the N- and C-terminus) and purified the expressed protein using Ni-NTA agarose beads (S3 Fig). The purified protein was subjected to mass spectrometry (MS). The MS data perfectly matched the findings obtained via alanine substitution and CIP-treatment analysis, confirming S50, S58, S62, S87, and S92 as the phosphorylated sites in Def (Fig 5F and S4 Fig).

### Simultaneous Phosphorylations at S58 and S62 or at S87 and S92 Are Necessary for Def to Promote Liver Development

**Fig 6:**
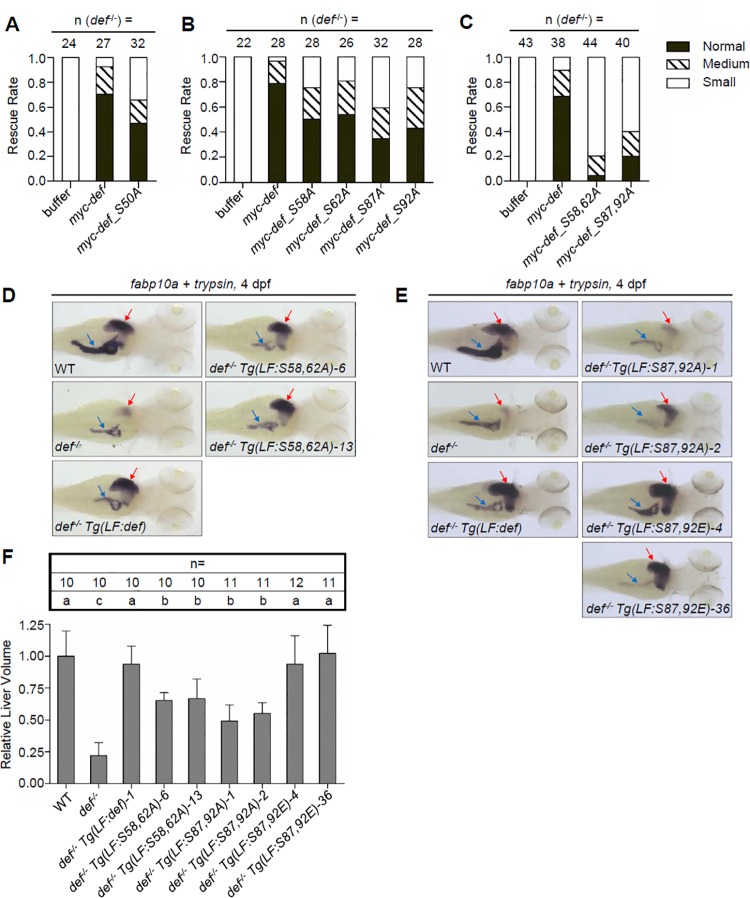
Simultaneous phosphorylations at S58 and S62 or at S87 and S92 are necessary for Def to promote liver development. **(A–C)** Histogram showing the rescue rate of liver development in *def*^*-/-*^ embryos injected with Myc-tagged *def* and *def_S50A* **(A)**, *def_S58A*, *def_S62A*, *def_S87A*, and *def_S92A* **(B)**, or *def_S58*,*62A* and *def_S87*,*92A* mRNA **(C)**. Injected embryos at 3.5 dpf were examined by WISH using the *fabp10a* probe (numbers of *def*^*-/-*^ embryos examined are shown above the bar), and the sizes of liver were classified into small, medium, and normal three groups. **(D, E)** WISH analysis of liver (red arrow) and exocrine pancreas (blue arrow) development in different transgenic fishes at 4 dpf using the *fabp10a* (for liver) and *trypsin* (for exocrine pancreas) probes simultaneously. **(F)** Each transgenic fish line was crossed to the reporter line *Tg(fabp10a*:*dsRed; elastase*:*GFP)*; background and liver volumes were obtained by 3-D reconstruction of the DsRed signal acquired using a confocal microscope. Total number of embryos used for each genotype was shown above each bar. Data are presented as means with SD. Columns with no common letter are significantly different (*p* < 0.001, one-way ANOVA with Tukey’s post hoc test). Underlying data for A, B, C, and F are provided in S1 Data.

We reported previously that loss of function of Def in zebrafish results in a phenotype with a small liver, a small pancreas, and thinned intestines [11]. Phosphorylation is a reversible process and plays important roles in the regulation of protein function. To study whether the Def phosphorylation status is important for protein function in liver development, we injected *def* and each type of *def*^*m*^ (*m* stands for any phosphorylation site mutation) mRNA into one-cell stage *def*^*-/-*^ zebrafish embryos and monitored liver development by whole-mount in situ hybridisation (WISH) using an *fabp10a* probe (a liver-specific marker) [29]. Rescue rates of Def single phosphorylation site mutants (Def_S50A, Def_S58A, Def_S62A, Def_S87A, and Def_S92A) were between 59% (Def_S87A) and 75% (Def_S58A and Def_S92A). This is slightly lower than those of WT Def (Fig 6A and 6B). Alignment of Def amino acid sequences showed that S58 and S62 are conserved in humans, mice, and zebrafish, whereas S87 and S92 are specific to zebrafish (S5 Fig). Considering the positional relationship between S58 and S62 and between S87 and S92, we generated two double mutants: Def_S58,62A (both S58 and S62 substituted with A) and Def_S87,92A (both S87 and S92 substituted with A). We found that the rescue rates of *def_S58*,*62A-* and *def_S87*,*92A* mRNA injection declined drastically to approximately 20% and 40%, respectively (Fig 6C), suggesting that simultaneous phosphorylation at S58 and S62 or at S87 and S92 might play a role in liver development.

We showed previously that Def functions as a cell autonomous factor in the development of the digestive organs in zebrafish by studying the *Tg(fabp10a*:*def)* transgenic fish, which expresses the zebrafish *def* gene driven by the liver specific promoter *fabp10a* [11,20]. This allows us to study the function of Def phosphorylation in liver development. For this purpose, we generated the transgenic fish *Tg(fabp10a*:*def_S58*,*62A)* and *Tg(fabp10a*:*def_S87*,*92A)*, which express the corresponding Def^m^ proteins, specifically in the liver, driven by the *fabp10a* promoter (S6 Fig) [20]. We also generated the *Tg(fabp10a*:*def_S87*,*92E)* transgenic fish to mimic the constitutive phosphorylation of S87 and S92 (S6 Fig). Two independent lines were obtained for each of the expression constructs (S6A Fig). WISH analysis revealed that, as observed in the *Tg(fabp10a*:*def)* transgenic fish [20], the *def*^*m*^ transcripts were expressed highly and specifically in the liver of all transgenic fish at 4 dpf (S6B and S6C Fig). These transgenic lines (including *Tg(fabp10a*:*def)*) were crossed with *def*^*hi429/+*^ (*def*^*+/-*^) to get the *def*^*+/-*^ *Tg(fabp10a*:*def)* and *def*^*+/-*^ *Tg(fabp10a*:*def*^*m*^*)* lines. Immunostaining of Def in *def*^*-/-*^ *Tg(fabp10a*:*def)* and *def*^*-/-*^ *Tg(fabp10a*:*def*^*m*^*)* 4-dpf embryos revealed both an over-expression of Def and Def^m^ localisation in the nucleolus (S7 Fig), which suggests that phosphorylation at the N-terminus does not affect Def nucleolar localisation.

We then compared liver and pancreas development among WT, *def*^*-/-*^, *def*^*-/-*^ *Tg(fabp10a*:*def)*, and each of the *def*^*-/-*^ *Tg(fabp10a*:*def*^*m*^*)* embryos at 4 dpf by WISH using *fabp10a* and the pancreatic marker *trypsin*. We observed that the small liver phenotype in *def*^*-/-*^ was fully restored to normal in *def*^*-/-*^ *Tg(fabp10a*:*def)* but was only partially rescued in *def*^*-/-*^ *Tg(fabp10a*:*S58*,*62A)* and *def*^*-/-*^ *Tg(fabp10a*:*S87*,*92A)* embryos at 4 dpf (Fig 6D and 6E). This is consistent with the results obtained with corresponding mRNA injections (Fig 6A–6C). Because liver size was fully restored in *def*^*-/-*^ *Tg(fabp10a*:*S87*,*92E)*, it is apparent that simultaneous phosphorylation at S87 and S92 is necessary for normal liver development (Fig 6E). In contrast, compared to WT, all genotypes in the *def*^*-/-*^ background still exhibited a small pancreas as expected, consistent with the fact that Def functions as a cell autonomous factor (Fig 6D and 6E).

To obtain a more precise measurement of the liver size, we crossed the *def*^*+/-*^, *def*^*+/-*^ *Tg(fabp10a*:*def)*, and *def*^*+/-*^ *Tg(fabp10a*:*def*^*m*^*)* fish into the *Tg(fabp10a*:*dsRed; elastase*:*GFP)* background (with the fluorescent protein DsRed expressed specifically in hepatocytes and GFP in exocrine pancreatic cells) [30]. We computed the liver volumes of 4-dpf embryos in each genotype by three-dimensional reconstruction and found that the liver sizes in *def*^*-/-*^ *Tg(fabp10a*:*def)* and the two *def*^*-/-*^ *Tg(fabp10a*:*S87*,*92E)* lines were not significantly different from a WT liver. However, the average liver volume in *def*^*-/-*^ *Tg(fabp10a*:*S58*,*62A)* and *def*^*-/-*^ *Tg(fabp10a*:*S87*,*92A)* was approximately 49% (*def*^*-/-*^ *Tg(fabp10a*:*S87*,*92A)-1*) to 66% (*def*^*-/-*^ *Tg(fabp10a*:*S58*,*62A)-13*) of that observed in WT fish (*p* < 0.001) (Fig 6F). These results show that Def phosphorylation promotes liver development.

### Simultaneous Phosphorylations at S58 and S62 or at S87 and S92 Are Necessary for Def to Promote Cell Cycle Progression

The liver development defect in *def*^*-/-*^ is due to cell cycle arrest in both the G1 to S and G2 to M progressions but is not due to cell apoptosis [11]. Because Def_S58, 62A, and Def_S87,92A only partially rescue the liver size of *def*^*-/-*^, we carried out a terminal deoxynucleotidyl transferase dUTP nick-end labelling (TUNEL) assay and found no abnormal apoptotic activity in the liver of *def*^*-/-*^ *Tg(fabp10a*:*S58*,*62A)* and *def*^*-/-*^ *Tg(fabp10a*:*S87*,*92A)*, thus excluding the possible contribution of cell apoptosis in the partial rescue phenotype (S8 Fig).

**Fig 7:**
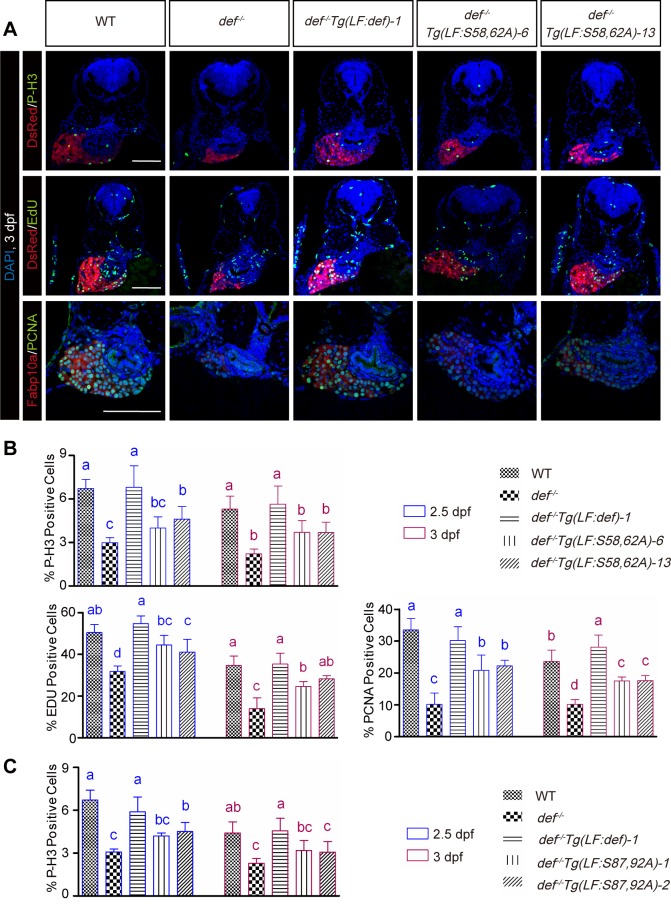
Simultaneous phosphorylations at S58 and S62 or at S87 and S92 are necessary for Def to promote cell cycle progression. **(A, B)** Representative images of P-H3, EdU, and PCNA immunostaining in *def*^*-/-*^ *Tg(LF*:*S58*,*62A)-6* or *-13* two lines and relavant control lines **(A)**. Scale bar: 100 μm. Hepatocytes were marked by showing DsRed fluorescence produced by the *Tg(fabp10a*:*dsRed; elastase*:*GFP)* transgenic fish (images for P-H3 and Edu staining) or by an Fabp10a antibody (images for PCNA staining). Nuclei were stained by DAPI. Statistical data for the ratio of P-H3, EdU, and PCNA positive cells in each genotype at 2.5 and 3 dpf were shown, respectively. **(B)** Data are presented as means with SD. Columns with no common letter are significantly different (*p* < 0.05, one-way ANOVA with Tukey’s post hoc test). **(C)** Histogram showing the statistical data for the ratio of P-H3 positive cells in *def*^*-/-*^ *Tg(LF*:*S87*,*92A)-1* or *-2* two lines and relavant control lines. Underlying data for B and C are provided in S1 Data.

Next, we performed immunostaining for phosphorylated histone 3 (P-H3), a marker for proliferating cells at the M phase, and recorded the number of P-H3-positive cells versus the total number of liver cells counted at 2.5 and 3 dpf. The result showed that the percentages of P-H3 positive cells were higher in *def*^*-/-*^ *Tg(fabp10a*:*def_S58*,*62A)-6* (4.0% at 2.5 dpf and 3.6% at 3 dpf) and *def*^*-/-*^ *Tg(fabp10a*:*def_S58*,*62A)-13* (4.5% at 2.5 dpf and 3.6% at 3 dpf) than those in *def*^*-/-*^ (3.0% at 2.5 dpf and 2.5% at 3 dpf). However, the highest percentages of P-H3 positive cells were observed in WT and *def*^*-/-*^ *Tg(fabp10a*:*def)* (6.6% in WT and 7.1% in *def*^*-/-*^ *Tg(fabp10a*:*def)* embryos at 2.5 dpf, and 5.2% in WT and 5.5% in *def*^*-/-*^ *Tg(fabp10a*:*def)* embryos at 3 dpf) (Fig 7A and 7B, P-H3 panel; S2 and S3 Tables). These data showed that simultaneous phosphorylation at S58 and S62 promoted a G2 to M transition.

EdU labelling is routinely used to detect S-phase cells based on the incorporation of EdU into newly synthesised DNA. We analysed EdU incorporation by recording the number of EdU-positive cells versus the total number of liver cells counted in WT, *def*^*-/-*^, *def*^*-/-*^ *Tg(fabp10a*:*def)*, *def*^*-/-*^ *Tg(fabp10a*:*def_S58*,*62A)-6*, and *def*^*-/-*^ *Tg(fabp10a*:*def_S58*,*62A)-13*. Statistical analysis showed that the ratios of EdU-positive cells were higher in *def*^*-/-*^ *Tg(fabp10a*:*def_S58*,*62A)-6* (43% at 2.5 dpf and 25% at 3 dpf) and *def*^*-/-*^ *Tg(fabp10a*:*def_S58*,*62A)-13* (42% at 2.5 dpf and 28% at 3 dpf) than those in *def*^*-/-*^ (32% at 2.5 dpf and 13% at 3 dpf). However, the highest ratios of EdU-positive cells were observed in WT and *def-/-Tg(fabp10a*:*def)* (51% in WT and 54% in *def*^*-/-*^ *Tg(fabp10a*:*def)* embryos at 2.5 dpf and 35% in WT and 35% in *def*^*-/-*^ *Tg(fabp10a*:*def)* embryos at 3 dpf) (Fig 7A and 7B, EdU panel; S2 and S3 Tables). PCNA is another proliferative marker [31,32]. To our surprise, most of the liver cells in the 2.5- and 3-dpf embryos examined for all genotypes appeared to be PCNA-positive (Fig 7A, PCNA panel). However, upon closer examination at a higher magnification, we noticed that the PCNA-positive cells fell into two categories: (i) those with enlarged nuclei with distinct bright foci that were taken as cells in active DNA synthesis (S9 Fig) and (ii) those with an even PCNA signal distribution or less prominent PCNA bright foci, which were taken as cells in non-active DNA synthesis [31,32]. On the basis of the above criteria, we compared the ratios of PCNA-positive cells (in active DNA synthesis) among the different genotypes and found that *def*^*-/-*^ *Tg(fabp10a*:*def_S58*,*62A)-6* and *def*^*-/-*^ *Tg(fabp10a*:*def_S58*,*62A)-13* were consistently less effective in promoting cell cycle progression (Fig 7A and 7B, PCNA panel; S2 and S3 Tables). Therefore, simultaneous phosphorylation at S58 and S62 also promotes a G1-to-S transition. Taken together, our results suggested that phosphorylation at S58 and S62 is necessary for Def to perform its complete function in promoting cell cycle progression.

The P-H3 staining patterns for the *def*^*-/-*^ *Tg(fabp10a*:*S87*,*92A)* line are similar to those of *def*^*-/-*^ *Tg(fabp10a*:*S58*,*62A)*. At 2.5 and 3 dpf, the percentages of P-H3–positive cells were higher in *def*^*-/-*^ *Tg(fabp10a*:*S87*,*92A)-1* (4.2% at 2.5 dpf and 3.2% at 3 dpf) and *def*^*-/-*^ *Tg(fabp10a*:*S87*,*92A)-2* (4.4% at 2.5 dpf and 3.0% at 3 dpf) than those in *def*^*-/-*^ (3.1% at 2.5 dpf and 2.3% at 3 dpf). However, the highest percentages of P-H3–positive cells were observed in WT and *def*^*-/-*^ *Tg(fabp10a*:*def)* (6.5% in WT and 5.9% in *def*^*-/-*^ *Tg(fabp10a*:*def)* embryos at 2.5 dpf, and 4.4% in WT and 4.4% in *def*^*-/-*^ *Tg(fabp10a*:*def)* embryos at 3 dpf) (Fig 7C and S4 Table). Therefore, simultaneous phosphorylation at S87 and S92 was also necessary for Def to promote cell cycle progression.

**Fig 8:**
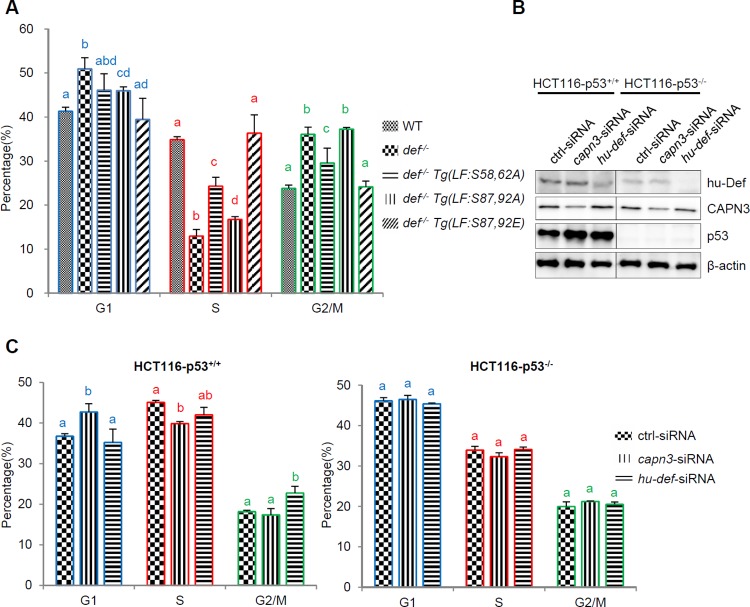
Def phosphorylation modulates its role in cell cycle in zebrafish and knockdown of CAPN3 or Def in cultured human cells causes a p53-dpendent cell cycle arrest. **(A)** Histogram showing flow cytometry analysis of the ratio of G1, S, and G2/M phases of liver cells collected from 8-dpf zebrafish of different genotypes as shown. **(B)** Western blot showing the effect of CAPN3 or hu-Def knockdown on p53 protein level in HCT116-p53^+/+^ or HCT116-p53^-/-^ cells at 24 h post treatment. β-actin: loading control. **(C)** Histogram showing flow cytometry analysis of the ratio of cells at G1, S, and G2/M phases in HCT116-p53^+/+^ (left panel) and HCT116-p53^-/-^ cells (right panel) treated with ctrl-siRNA, *capn3*-siRNA, or *hu-def*-siRNA at 24 h post treatment. Data are presented as means with SD. Columns with no common letter are significantly different (*p* < 0.05). Underlying data for A and C are provided in S1 Data.

To further characterise the effect of Def phosphorylation on cell cycle progression, we dissected the liver bud (with hepatocytes genetically labelled by expressing DsRed) from embryos of different genotypes at 8 dpf and dissociated the liver cells (containing >94% hepatocytes based on counting the number of DsRed-positive cells) for DNA content determination by flow cytometry analysis (S10A–S10D Fig). The result showed that the cell cycle in *def*^*-/-*^ was arrested at both the G1 and G2/M phases (an average of 41% G1 and 24% G2/M cells in WT versus 51% G1 and 36% G2/M cells in *def*^*-/-*^, *p* < 0.005) (Fig 8A and S10E Fig). This was consistent with our analysis of cell cycle markers by immunostaining (Fig 7). The G2/M arrest in *def*^*-/-*^ was partially restored in *def*^*-/-*^ *Tg(fabp10a*:*S58*,*62A)* (30% of G2/M cells, *p* < 0.05), whereas the G1 arrest was partially restored in *def*^*-/-*^ *Tg(fabp10a*:*S87*,*92A)* (46% G1 cells, *p* < 0.05). Notably, although fewer than those in the WT (35%), there were more cells at the S phase in both *def*^*-/-*^ *Tg(fabp10a*:*S58*,*62A)* (24%) and *def*^*-/-*^ *Tg(fabp10a*:*S87*,*92A)* (17%) than in *def*^*-/-*^ (13%) (Fig 8A and S10E Fig). As expected, the cell cycle was fully rescued in *def*^*-/-*^ *Tg(fabp10a*:*S87*,*92E)* (Fig 8A and S10E Fig).

We showed previously that the knockdown of CAPN3 resulted in up-regulation of p53 in HepG2 cells (p53 WT) but not in H1299 cells (p53 null) [9,17]. We also showed that Def and Capn3 form a complex in the nucleolus (Fig 2), and p53 is one of the targets of the Def-Capn3 pathway (Fig 1). To further investigate the role of Def and Capn3 in cell cycle progression in human cells, we knocked down the expression of the endogenous hu-Def or CAPN3 in HCT116-p53^+/+^ (p53 WT) and HCT116-p53^-/-^ (p53 null) cells (Fig 8B). Consistent with our previous report on other p53 wild-type human cells [17,33], knockdown of either hu-Def or CAPN3 in HCT116-p53^+/+^ also caused up-regulation of the p53 protein level (Fig 8B). Flow cytometry analysis revealed that knockdown of the endogenous CAPN3 arrested the cell cycle at the G2 phase in HCT116-p53^+/+^ but had no effect on HCT116-p53^-/-^ cells (Fig 8C and S11 Fig). Knockdown of the endogenous hu-Def also caused cell cycle arrest but at the G2/M phase instead of the G1 phase in HCT116-p53^+/+^ (Fig 8C and S11 Fig). These results suggested that for cultured human cells: (1) Def- or CAPN3-mediated cell cycle progression strongly correlates with the activation of the p53 pathway and (2) because, in addition to forming the Def-CAPN3 complex, Def is also a component of the ribosome small subunit (SSU) processome and CAPN3 also functions in the cytoplasm, this probably explains why the cells are arrested at different stages of the cell cycle after knockdown of their expression.

### Simultaneous Phosphorylations at S87 and S92 Facilitate the Nucleolar Localisation of CAPN3

**Fig 9:**
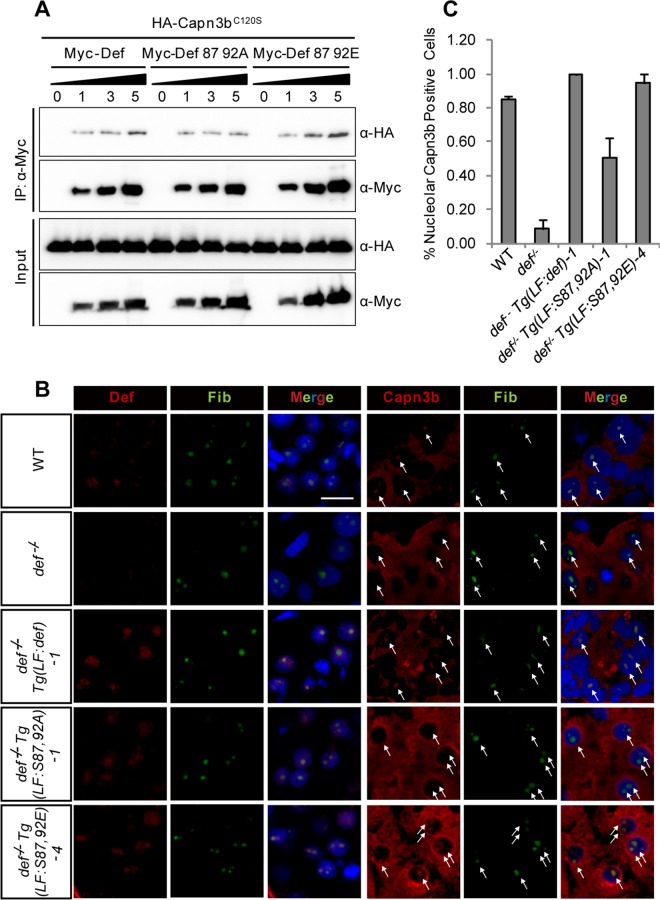
Simultaneous phosphorylations at S87 and S92 are necessary for the nucleolar localisation of Capn3b. **(A)** Co-IP analysis of the interaction between Capn3b and Def, Def_S87,92A, or Def_S87,92E. The 293T cells were transfected with *HA-capn3b*^*C120S*^ plasmid alone or in combination with *Myc-def*, *Myc-def_S87*,*92A*, or *Myc-def_S87*,*92E* plasmid in three different ratios (*HA-capn3b*:*Myc-def*^*m*^ ratio: 1:1, 1:3, or 1:5), and total protein was extracted 2 d after transfection. Antibodies against the HA-tag (α-HA) or Myc-tag (α-Myc) were used in western blotting. **(B, C)** Co-immunostaining of the endogenous Def and Capn3b in the liver of 6.5-dpf zebrafish of different genotypes. The percentage of Capn3b-positive cells in different genotypes in **(B)** was summarized in **(C)**. Nucleoli are indicated by arrows. Scale bar: 10 μm. Underlying data for C are provided in S1 Data.

We have shown that the nucleolar localisation of Capn3b/CAPN3 is Def-dependent (Fig 3A and 3B) and that *Tg(fabp10a*:*S87*,*92E)* rescues and *Tg(fabp10a*:*S87*,*92A)* partially rescues the small liver phenotype conferred by *def*^*-/-*^ (Fig 6E). To determine a correlation between Def phosphorylation and the nucleolar localisation of Capn3b, we first analysed the response of Capn3b to different dosages of Def, Def_S87,92A, and Def_S87,92E via Co-IP assay. The results showed that both WT Def and Def_S87,92E exhibited a dose-dependent interaction with Capn3b in an overexpression system, whereas Def_S87,92A interacted with Capn3b in a less efficient manner (Fig 9A and S2C Fig). Next, we analysed the subcellular localisation of Capn3b in the *def*^*-/-*^ *Tg(fabp10a*:*S87*,*92A)* and *def*^*-/-*^ *Tg(fabp10a*:*S87*,*92E)* fish at 6.5 dpf by co-immunostaining Fib and Capn3b in comparison with that in the *def*^*-/-*^ *Tg(fabp10a*:*def)* fish. We found that the nucleolar localisation of Capn3b was restored in the hepatocytes of *def*^*-/-*^ *Tg(fabp10a*:*S87*,*92E)* fish as observed for *def*^*-/-*^ *Tg(fabp10a*:*def)* but was only partially restored in *def*^*-/-*^ *Tg(fabp10a*:*S87*,*92A)* (Fig 9B and 9C, S1 Table). Therefore, we concluded that the translocation of CAPN3/Capn3b into the nucleolus is Def-dependent and that Def phosphorylation at S87 and S92 facilitates the nucleolar localisation of Capn3b.

### Simultaneous Phosphorylations at S58 and S62 or at S87 and S92 Are Essential for Def to Promote p53 Degradation in the Nucleolus

**Fig 10:**
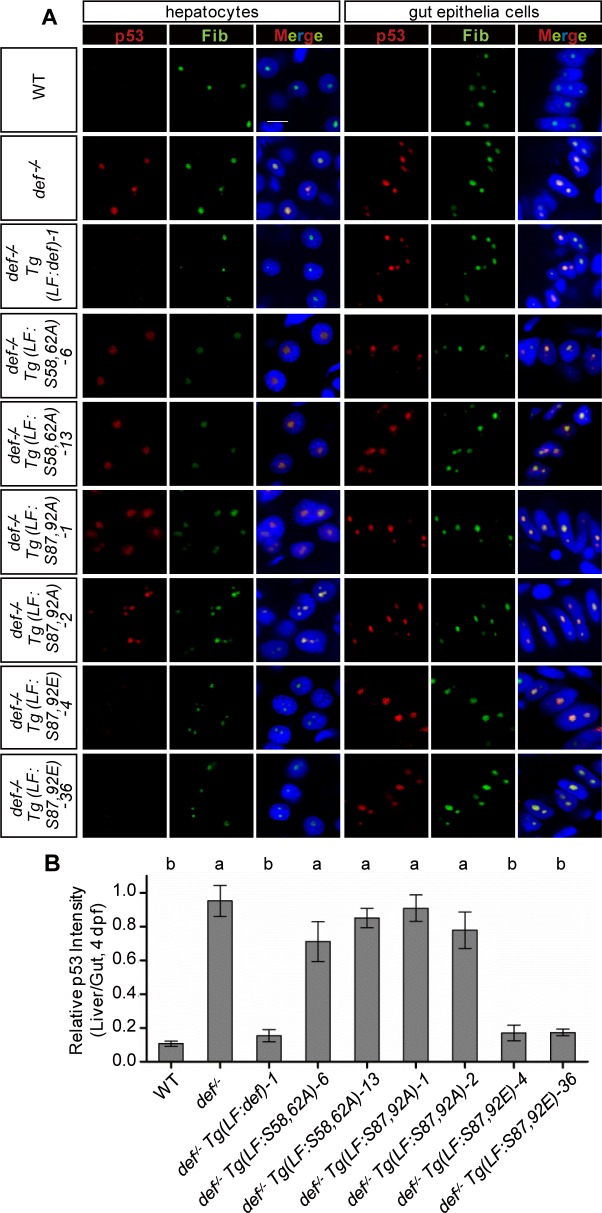
Simultaneous phosphorylations at S58 and S62 or at S87 and S92 are essential for Def to mediate p53 degradation in the nucleoli. **(A)** Co-immunostaining of p53 (in red) and Fib (in green) in different genotypes as shown. DAPI was used to stain the nuclei (blue). Scale bar: 5 μm. **(B)** Statistical data showing the fold change of p53 signal intensity for hepatocytes versus intestinal epithelial cells in **(A)**. Data are presented as means with SEM. Columns with no common letter are significantly different (*p* < 0.001, one-way ANOVA with Tukey’s post hoc test). Underlying data for **(B)** are provided in S1 Data.

In the *def*^*-/-*^ mutant, the elevated p53 protein levels accumulated in the nucleolus [17]. We were intrigued to determine the p53 status in the liver of the *def*^*-/-*^ *Tg(fabp10a*:*def)* transgenic fish. To do this, we performed co-immunostaining of p53 and Fib in 4-dpf WT, *def*^*-/-*^, and *def*^*-/-*^ *Tg(fabp10a*:*def)* embryos followed by quantification of their signal intensities in the liver and gut epithelium, respectively, in each section. As expected, although p53 was almost undetectable in the nucleolus of WT, it was accumulated in the nucleolus of *def*^*-/-*^ (Fig 10A, S5 Table). In *def*^*-/-*^ *Tg(fabp10a*:*def)*, p53 was no longer observed in the nucleolus of the hepatocytes but remained prominent in the nucleolus of gut epithelial cells (Fig 10A, S5 Table). This is consistent with the phenotypic rescue of liver development but not the development of other digestive organs in *def*^*-/-*^ *Tg(fabp10a*:*def)* (Fig 6D and 6E) [20]. Similarly, we examined the p53 status in each of the *def*^*-/-*^ *Tg(fabp10a*:*def*^*m*^*)* embryos at 4 dpf and found that p53 was still highly accumulated in the nucleolus of the hepatocytes in *def*^*-/-*^ *Tg(fabp10a*:*S58*,*62A)* and *def*^*-/-*^ *Tg(fabp10a*:*S87*,*92A)* but not in *def*^*-/-*^ *Tg(fabp10a*:*S87*,*92E)* (Fig 10A, S5 Table). Quantitative comparison of the nucleolar p53 signal intensities between the liver and gut showed that Def_S58,62A and Def_S87,92A were compromised in the promotion of p53 degradation in the nucleolus (Fig 10B).

### Def Promotes Liver Development Only in Part through the p53 Pathway

**Fig 11:**
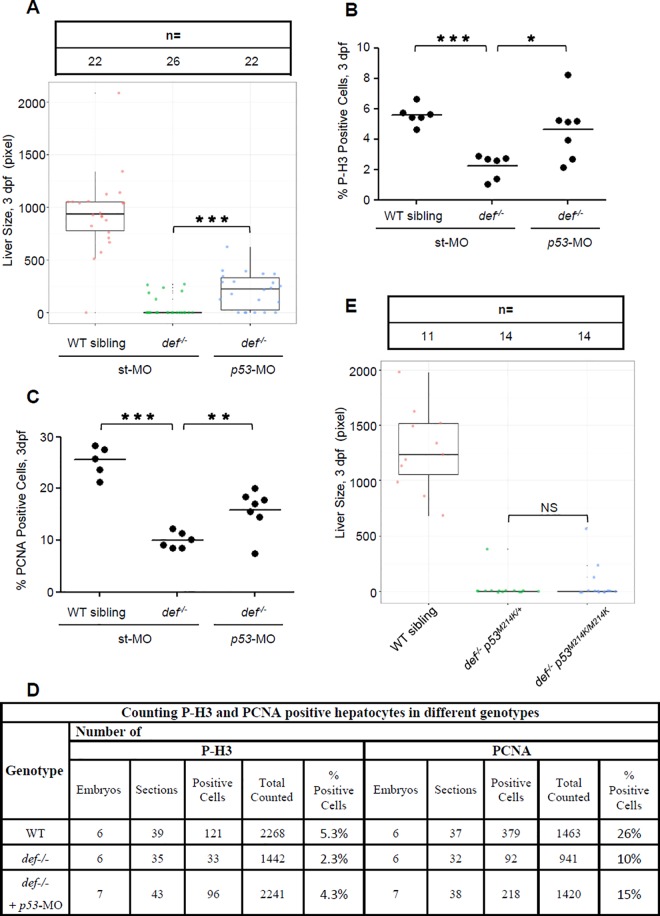
Def regulates liver development only in part through the p53 pathway. **(A–D)** WISH analysis of liver development **(A)** and histogram showing the statistical data for the ratio of P-H3- **(B)** or PCNA- **(C)** positive cells in *def*^*-/-*^ embryos injected with *p53*-specific morpholino mixes (ATG-MO plus spl-MO) at 3 dpf. Number of embryos used for sectioning and number of sections used for counting P-H3- and PCNA-positive hepatocytes in different genotypes in **(B)** and **(C)** was shown in **(D)**. **(E)** WISH analysis of liver development in the *def*^*-/-*^ *p53*^*M214K*^ double mutant at 3dpf. The *fabp10a* probe was used for WISH. Liver size is measured by liver area marked by the *fabp10a*-positive signal. In the quartile boxplot, each dot represents the liver size of an individual embryo. st-MO, standard control morpholino; ATG-MO, morpholino targeting the translation start site (ATG) of *p53*; spl-MO, morpholino targeting the splicing junction of exon 5 and intron 5 of *p53*. In **(A)** and **(E)**, total number of embryos used for each genotype is shown on the top of the graph. Underlying data for (A), (B), (C), and (E) are provided in S1 Data.

Because p53 is a target of the Def-Capn3 pathway, one scenario is that loss of function of p53 might rescue liver development in *def*^*-/-*^. We previously showed that the knockdown of p53 expression only partially rescued pancreas development in *def*^*-/-*^, which led us to propose that Def promotes digestive organ development in part through the p53 pathway. To further confirm this hypothesis, we co-injected p53-MO^ATG^ (to block the translation of both maternal and zygotic p53 mRNA) and p53-MO^spl^ (to block the maturation of zygotic p53 mRNA) [11] into *def*^*-/-*^ and its siblings (WT or heterozygotes) (S12A Fig). The injected embryos were first examined for the liver development. The results showed that, consistent with our previous report on the pancreas, liver development was partially rescued in a portion of the embryos by knockdown of p53 expression (Fig 11A). Bhmt and Fabp10a are two markers for hepatocytes [2,19]. Co-immunostaining of P-H3 with Bhmt or PCNA with Fabp10a allowed us to count P-H3- or PCNA-positive hepatocytes. We found that, in the *def*^*-/-*^ embryos co-injected with p53-MO^ATG^ and p53-MO^spl^, the percentages of P-H3-positive hepatocytes were higher in morphants (4.3%) than in *def*^*-/-*^ (2.3%). Similarly, the pecentages of PCNA-positive hepatocytes were higher in the morphants (15%) than in *def*^*-/-*^ (10%) (Fig 11B–11D and S12B Fig). These results suggest that the cell cycle arrest of the hepatocytes in the *def*^*-/-*^ embryos was partially rescued after co-injection of p53-MO^ATG^ and p53-MO^spl^. Next, we crossed *def*^*-/-*^ to the p53 mutant (p53^M214K^ transcriptional inactive form, carrying a M^214^ to K^214^ substitution in the p53 DNA-binding domain) and obtained the *def*^*-/-*^ *p53*^*M214K*^ double mutant. Surprisingly, analysis of the liver development using an *fabp10a* probe showed no significant difference in liver sizes between the *def*^*-/-*^ single and *def*^*-/-*^ *p53*^*M214K*^ double mutant (Fig 11E), indicating that, although p53^M214K^ lost its ability to activate the expression of certain p53-response genes, it probably was still a functional protein (having either partial loss or gain offunction), as previously reported [34].

## Discussion

The nucleolus is a peculiar subnuclear structure that houses several hundred to a thousand proteins. Increasingly, data have shown that the nucleolus is not only a site for the assembly of ribosomal subunits but is also highly responsive to extrinsic or intrinsic stress signals to regulate cell behaviour. This latter function is largely due to the great diversity of functional proteins localised within the nucleolus. In this report, we showed that Def and CAPN3 form a complex in the nucleolus and that Def determines the nucleolar localisation of CAPN3. We further showed that the function of the Def-CAPN3 protein degradation pathway is regulated by Def phosphorylation at five Ser residues: S50, S58, S62, S87, and S92. Specifically, simultaneous phosphorylation at S58 and S62 or at S87 and S92 is not only important for Def in the mediation of p53 degradation in the nucleolus but is also essential for normal transition from G1 to S and from G2 to M during cell proliferation. Our findings firmly specify a key role of the nucleolar Def-CAPN3 protein degradation pathway in the cell cycle progression during organogenesis, thus establishing a novel biological role for the nucleolus—the “old factory.”

Def was first identified as an important factor in promoting the growth of digestive organs in zebrafish [11]. Further studies revealed that Def is a component of the small subunit processome [12–14,20]. Our studies showed that Def cooperated with CAPN3 to mediate protein degradation and that one of the substrates of the Def-CAPN3 pathway is p53 [17], and we proved that p53 is a direct target of CAPN3. We also showed that CAPN3 forms a complex with Def, and that this complex is essential for CAPN3 nucleolar localisation. These facts suggest that Def likely functions as a carrier protein to ferry CAPN3 to the nucleolus. Interestingly, the human p53^R175H^ mutant protein is CAPN3 insensitive. This is consistent with our previous report on the insensitivity of p53^R143H^ to Def-mediated p53 degradation in zebrafish. It is probable that the R175 mutation is close to the CAPN3 recognition motif (188–198 AA) in p53, and that the R175H mutation alters the conformation in the CAPN3 recognition motif in p53 [35] that makes p53^R175H^ resistant to the CAPN3 enzymatic activity. This implies that patients who carry the *p53*^*R175H*^ mutation can potentially accumulate a high level of non-degraded p53 in the nucleoli. Indeed, by analysing the distribution of p53 proteins in three different types of breast cancer cells, we found that p53^R175H^ appeared to accumulate in the nucleolar fraction (S13A and S13B Fig). We also analysed the status of the p53^R175H^ mutation in cancer samples by searching the Catalogue of Somatic Mutations in Cancer database (http://cancer.sanger.ac.uk/cosmic) and identified the mutation at p53^R175^ as one of the hotspots for cancer mutation (with 1,260 tumour samples harbouring the R175 substitution mutant) (S13C Fig). Furthermore, the p53^R175H^ mutation ranks first among the p53 substitution mutations in cancer samples (S13D Fig), and p53^R175H^ is not specific for any cancer type but is more frequently observed in large intestine, breast, haematopoietic, lymphoid, and ovary tumour samples (S13E Fig).

Protein function is often modulated by the modification of phosphorylation status [36]. Through mutant mRNA injection and detailed analysis of transgenic fish that express different mutant Def^m^ proteins, specifically in the liver, we showed that simultaneous phosphorylation of the S58 and S62 pair and the S87 and S92 pair regulates Def function, particularly its role in the mediation of nucleolar p53 degradation, cell cycle progression, and nucleolar localisation of Capn3b. Thus, we propose that the function of Def phosphorylation is probably to provide a microenvironment that is favourable for the enzymatic activity of CAPN3. Alternatively, phosphorylation is important for Def to interact with other nucleolar partners that are essential for the activity of CAPN3. In summary, we propose that Def not only serves as a carrier to ferry CAPN3 to the nucleolus but also acts as a scaffold protein to provide a setting for the enzymatic activity of CAPN3 on the target protein. Interestingly, protein sequence alignment showed that S58 and S62 are conserved among zebrafish, mice, and humans, which implies that these two sites are also likely to be important in mammals. In contrast, S87 and S92 in zebrafish are not conserved in mammals (corresponding to E91 and E96, respectively, in both human and mouse Def). The likely scenario is that during evolution, S87 and S92 in lower vertebrates was converted to E91 and E96 in mammals, suggesting that in the lower vertebrates, these two amino acids need to be modified by phosphorylation to fulfil their biological function, whereas in mammals, these two amino acids mimic the constitutive active form of the zebrafish Def as shown in the *Tg(fabp10a*:*def_S87*,*92E)* transgenic fish. Regarding the role of Def in liver development, we argue that because Def not only teams up with Capn3 to form the Def-Capn3 pathway but also is a component of the ribosomal small subunit processome (for the biogenesis of ribosome), both of these functions of Def might contribute to promoting digestive organ growth, including liver. This would explain the partial rescue of the digestive organ development in *def*^*-/-*^ when the expression of p53 is knocked down, because there might be other substrates of the Def-Capn3 pathway, which are essential to regulate liver development, or the function of Def in ribosome biogenesis is also necessary for the growth of digestive organs, including liver.

Many questions remain to be answered about this novel nucleolar protein degradation pathway. For example, it appears that one or more sites exist within the 190–377 AA region in Def that are also modified by phosphorylation. One impending task is to determine such site(s) in the region. It is now clear that Def is phosphorylated at multiple sites, so we must ask whether they are modified by single or multiple kinases and what these kinase identities are. Next, does Def phosphorylation form a regulatory feedback loop? If so, how is it regulated in response to different stimuli? Does Def-CAPN3 work with other partners to mediate p53 degradation in the nucleolus? In addition to p53, are there any other targets of the Def-CAPN3 pathway? Insights into these questions will be instrumental in understanding the biological function of the Def-Capn3 pathway.

## Materials and Methods

### Ethics Statement

All animal procedures were performed in full accordance to the requirement by “Regulation for the Use of Experimental Animals in Zhejiang Province.” This work was specifically approved by the Animal Ethics Committee in the School of Medicine, Zhejiang University (ETHICS CODE Permit NO. ZJU2011-1-11-009Y, issued by the Animal Ethics Committee in the School of Medicine, Zhejiang University).

### Zebrafish Lines and Maintenance

Zebrafish line *def*^*-/-*^ (*def*^*hi429*^) [33] was as described previously [11]. *Tg(fabp10a*:*def)-1* was generated as described [20]. To generate the *Tg(fabp10a*:*def*^*m*^*)* lines, each *fabp10a*:*def*^*m*^ was cloned into a pDB739 vector (modified from pminiTol2 obtained from Dr. Stephen Ekker) to obtain the *fabp10a*:*def*^*m*^*-miniTol2* plasmid. Each *fabp10a*:*def*^*m*^*-miniTol2* plasmid DNA was injected together with Tol2 transposase mRNA [37] into one-cell stage embryos to generate transgenic fish. Positives were scored by the primers *def*_Fw1644 (5ʹ-CCAGGTGTGCAGTAAAACCATT-3ʹ) and *def*_Rv2048 (5ʹ-GGCAACCCGTAGAAGATCAGA-3ʹ). Zebrafish were raised and maintained in accordance with the standard procedures [38].

### Plasmid Construction, siRNA, Morpholino

cDNA was cloned into pCS2^+^ vector. The human *CAPN3*, *myc*-tagged *p53*, *def*, *D1*, *D2*, *D3*, and *D4* were constructed as described [17]. The IS I domain is excluded in all human *CAPN3*-related plasmids. The *myc*-tagged *D9* and *D10* and *EGFP*-tagged *D9*, *D10*, *D14*, and *D15* constructs were generated as described [27].

All *def*-related, human *p53*, and *CAPN3* mutants were produced by site-directed mutagenesis using the primers listed in S6–S9 Tables. HA or MYC sequences were included in the primers to generate tagged constructs.

All siRNAs and morpholinos were used as previously described [11,17].

### Expression and Purification of CAPN3

*CAPN3* cDNA was cloned into the pFastBacTHB vector. SF-9 cells were cultured and infected by recombinant baculovirus according to the manufacturer’s instructions. At 50 h, the cells were collected by centrifugation at 1,000 *g* for 5 min at 4°C. The harvested cells were homogenized by sonication in 2 ml of buffer (20 mM Tris-HCl, 5 mM NaHCO3, 0.1 mM EGTA, 1 mM PMSF, 5 mM 2-mercaptoethanol, pH 7.5). After centrifugation, the supernatant was collected and incubated with Ni-NTA agarose beads (QIAGEN), washed twice, and eluted using 500 mM imidazole. The eluent was desalted by ultrafiltration with a pre-equilibration buffer (10 mM Tris-HCl, 5 mM EDTA, 1 mM DTT, pH 7.5).

### GST Pull-Down Assay

*GST-CAPN3* was cloned into the expression vector pGEX-6P-1. *His-Def*^*1-379*^ was obtained by PCR amplification using forward (5ʹ CGCGGATCCATGCATCATCACCACCACCATGGCAAACGCGGGAGCCGGAGC-3ʹ) and reverse (5ʹ-GCTCTAGAGTCACCCTCGAGGAGGCTGA-3ʹ) primers and cloned into pET30a. *E*. *coli* BL21 (DE3) was used for protein expression. The cells were sonicated in lysis buffer (20 mM Tris-Cl, 200 mM NaCl, 1 mM EDTA, 0.5% NP-40, pH8.0). The GST-CAPN3 lysate was incubated with GST-beads (Novagen) overnight and washed five times with lysis buffer. His-Def^1-379^ was retained by Ni-NTA agarose beads (QIAGEN), eluted by 500mM of imidazole, and desalted with lysis buffer. The GST beads were incubated with the purified His-Def^1-379^ for 8 h. The beads were washed six times and eluted with SDS lysis buffer.

### mRNA Synthesis and Western Blotting

mRNA was synthesised in vitro using mMESSAGE mMACHINE Kit (Ambion) according to the manufacturer’s instructions. A total of 0.2 ng of mRNA was injected into one-cell stage zebrafish embryos to overexpress the protein of interest. The embryos were first deyolked and lysed in SDS lysis buffer supplied with 1×Complete Protease Inhibitor Cocktail (EDTA-free, Roche). The protein samples were immediately used for western blot analysis or stored at -20°C. For western blot analysis, rabbit polyclonal antibody against Def (using the CLRLPDSPQRPEPDS peptide as antigen) and Capn3b was generated by Hangzhou Hua An Biotechnology Company (China). Anti-Myc tag antibody (9E10, No. 631206) was purchased from Clontech; GFP (B-2, No. sc-9996), p53 (DO-1, No. sc-126), (PAb240, No. sc-99) from Santa Cruz; CAPN3 (No. 38963), NPC (Mab414, No. ab24609), and p53 (PAb1620, No. ab16776) from Abcam; CAPN3 goat polyclonal antibody (No. COP-080048) from Cosmo Bio; GAPDH (EPR1977Y, No. 2251) from Epitomics, and GAPDH (5-E10, No. M1211), β-actin (R1207-1) from Hua An.

### Calf Intestinal Alkaline Phosphatase (CIP) Treatment

For CIP (NEB) treatment, the deyolked embryos were lysed in Tris lysis buffer (100 mM NaCl, 10 mM MgCl_2_, 1 mM dithiothreitol, 50 mM Tris-HCl, pH 7.9) supplied with 1×Complete Protease Inhibitor Cocktail (EDTA-free, Roche) and 0.2% NP40. Ten units of CIP were added to 40 μl supernatant and incubated at 37°C for 1 h. The reaction was stopped by adding 13 μl of SDS sample buffer before SDS-PAGE.

### Mass Spectrometry and Data Analysis

6×His tagged EGFP-D14 was derived from EGFP-D14-pCS2+ using the primers His-EGFP (5ʹ-CGGGATCCACCATGGCCCACCATCATCATCATCATGTGAGCAAGGGCGAGGAGCT-3ʹ, forward) and His-D14 (5ʹ-CGGAATTCTCAGTGGTGGTGGTGGTGGTGCTCTTCCTCGCTTTCTTCATCTTC-3ʹ, reverse). The amplified fragment was cloned into pCS2+ pre-digested with *BamHI* and *EcoRI* to generate the expression plasmid (6×His)-EGFP-D14-(6×His)-pCS2^+^. Then, 24 μg of plasmid DNA per plate was used to transfect 293T cells cultured in a 10 cm plate (three dishes were used). At 36 h after transfection, the total protein was extracted and (6×His)-EGFP-D14-(6×His) was purified using Ni-NTA agarose beads (Qiagen) according to the manufacturer’s instructions.

The SDS-PAGE gel containing approximately 50 μg (6×His)-EGFP-D14-(6×His) of protein was cut into 2- to 3-mm^3^ pieces and de-colored in 25 mM of ammonium bicarbonate/50% acetonitrile buffer. The samples were then incubated with 10 mM of DTT in 50 mM of ammonium bicarbonate at 56°C for 1h followed by 55 mM of iodoacetamide in 50 mM of ammonium bicarbonate in the dark for 45 min, followed by overnight digestion with trypsin (Sigma; enzyme-to-substrate ratio 1:50) in 25 mM ammonium bicarbonate at 37°C. Peptides were extracted from gel with buffers containing 5% trifluoroacetic acid and 50% acetonitrile by two rounds of ultrasonication. The liquid was freeze-dried by SpeedVac, and the peptides were resolubilised in 0.1% formic acid and filtered with a 0.45-μm centrifugal filter.

For the MS analysis, the resuspended peptides were analysed by LTQ Orbitrap Elite mass spectrometer (Thermo Fisher Scientific) coupled online to an Easy-nLC 1,000 (Thermo Fisher Scientific) in the data-dependent mode (the facility is located in the Institute of Genetics and Developmental Biology, CAS, Beijing). Peptides were separated by reverse phase LC with a 75 μm (ID) × 150 mm (length) analytical column packed with C18 particles of 5 μm in diameter. The mobile phases for the LC contain buffer A (2% ACN, 0.1% FA) and buffer B (98% ACN, 0.1% FA), and a linear gradient of buffer B from 3% to 30% for 90 min was used for separation. All MS measurements were performed in the positive ion mode. Precursor ions were measured in the Orbitrap analyser at 240,000 resolution (at 400 m/z) and a target value of 10^6^ ions. The 20 most intense ions from each MS scan were isolated, fragmented, and measured in the linear ion trap, with MSA neutral losses of m/z 98, 49, and 32.6. The CID normalised collision energy was set to 35.

The data were analysed using a prerelease version of Thermo Scientific Proteome Discoverer^TM^ software version 1.3. The proteome sequences for *Danio rerio* (including canonical and isoforms) from unitprot were used for a database search with the mass tolerance set to 0.05 Da. The maximum number of missed cleavages was set at two, with a minimum peptide length of six and a maximum peptide length of 144 amino acids. The false discovery rate was set at 0.01 for peptide and protein identification. Serine, threonine and tyrosine phosphorylation, cysteine carbamidomethylation, and methionine oxidation were included in the search as variable modifications.

### Whole-Mount RNA In Situ Hybridisation (WISH)

For WISH, digoxigenin (DIG, Roche Diagnostics) was used to label the *fabp10a*, *trypsin*, and *def* probes [11]. WISH was performed as previously described [11].

### Cryosectioning

The embryos were fixed in 4% PFA for 1 h at room temperature. After washing in PBST, the embryos were mounted with 1.5% agarose dissolved in 30% sucrose PBST and then equilibrated overnight in 30% sucrose PBST at 4°C. The blocks were mounted with OCT compound (Sakura). The sections were cut serially to a 12-μm thickness and collected on poly-L-lysine coated glass slides (Menzel, Cat. No. J2800AMNZ). The slides were used immediately for immunofluorescence or stored at -80°C.

### Immunofluorescence Staining

The cryosections were rehydrated with two washes of 1xPBS and then permeated with PBS plus 0.2% Triton X-100 for 5 min. After a brief wash with PBS, the sections were blocked by 20% goat serum in PBS plus 0.5% BSA. After a brief wash with PBS plus 0.5% BSA, the sections were incubated overnight with primary antibody at the desired concentration diluted in PBS plus 0.5% BSA at 4°C. After three washes with PBS plus 0.5% BSA, sections were incubated with secondary antibodies (1:400) and DAPI (1:500) in PBS plus 0.5% BSA for 1 h. After three washes with PBS plus 0.5% BSA, the sections were finally mounted in 80% glycerol and covered with coverslip for image acquisition. Def antibody (1:200) [11], antibodies against Fibrillarin (Abcam, ab4566, 1:600), P-H3 (Santa Cruz, sc-8656-R, 1:600), PCNA (Sigma, P8825, 1:1000), p53 (1:200) [17], and rabbit polyclonal antibody Fabp10a (1:1000), mouse monoclonal antibody Bhmt (1:450) generated by Hangzhou HuaAn Biotechnology Company, were used. An antigen retrieval process using sodium citrate buffer (10 mM citric acid, pH 6.0) was carried out before the blocking step for PCNA detection.

### TUNEL Assay

TUNEL assay was carried out following the procedures previously described [11] with the in situ cell death detection kit TMR red (Roche). Sections of WT embryos pretreated with DNase I were used as a positive control according to the manufacturer’s instructions.

### EdU Incorporation Assay

EdU (5-ethynyl-2ʹ-deoxyuridine, 1 nl, 10 mM) was injected into the anterior region of the yolk between the heart and liver of the embryos at 2.5 or 3 dpf. After incubation at 28.5°C for 2 h, the embryos were fixed in 4% PFA for 2 h before proceeding to cryo-section. Incorporated EdU was detected by Alexa Fluor 488 Azide (Life Technologies, A10266).

### Nucleoli Isolation

HepG2 cell nucleoli were isolated as previously described [3]. Briefly, the cells were homogenised in buffer A (10 mM Hepes, pH 7.9, 10 mM KCl, 1.5 mM MgCl_2_, and 0.5 mM DTT). After centrifugation at 218 *g* for 5 min at 4°C, the nuclear pellet was resuspended in S1 solution (0.25 M sucrose, 10 mM MgCl_2_) and cleared by centrifugation over a cushion of S2 solution (0.35 M sucrose, 0.5 mM MgCl_2_). The nuclei were resuspended in S2 solution and sonicated (Branson Sonifier, SLPe) for six 10-s bursts (with 30-s intervals between bursts) at 40% amplitude. The sonicated sample was layered onto S3 (0.88 M sucrose, 0.5 mM MgCl_2_) and centrifuged at 3,000 *g* for 10 min. The supernatant contained nucleoplasmic fractions. The nucleoli were resuspended with 0.5 ml of S2 solution, followed by centrifugation at 1,430 *g* for 5 min at 4°C.

The zebrafish liver nucleoli were isolated as previously described [39] with minor modification. The zebrafish livers were collected and homogenised in buffer A. After centrifugation at 500 *g* for 5 min at 4°C, the nuclear pellet was washed twice in buffer A. The pellet was then resuspended in zfS1 (0.5 M sucrose, 3 mM MgCl_2_) and sonicated for five 8-s bursts (with 15-s intervals between bursts) at 50% amplitude. The sonicated sample was layered onto zfS2 (1 M sucrose, 3 mM MgCl_2_) and centrifuged at 1,800 *g* for 10 min. The supernatant contained nucleoplasmic fractions.

### Flow Cytometry Analysis

Human cells were digested with trypsin and washed twice with PBS, then stained with PI (Propidium Iodide, Beyotime) according to the manufacturer’s instructions. Zebrafish embryos were raised to 8 dpf and fixed with 0.5% PFA at 4°C for 0.5 h. After washing with PBS, liver bud labeled by DsRed signal was dissected under a fluorescence microscope (Nikon, SMZ1000) using ultra micro tweezers; the non-liver tissues sticked to the liver were carefully removed in bright field using a fine needle. Liver cells were dissociated by treatment with 0.1% trypsin at 37°C for 20 min and were washed with PBS. Cells were stained with PI and subjected to flow cytometry analysis of the cell cycle using a BD FACS Calibur flow cytometer.

### Statistics

Statistical analyses were performed with the Student’s *t* test. * *p* < 0.05; ** *p* < 0.01; *** *p* < 0.001; N.S., no significant difference. To cope with the relative small sample sizes, we used the quartile boxplot to present our data on liver size measurement shown in Fig 11 [40]. The box plot was drawn by ggplot2 [41].

## Supporting Information

S1 Data Excel spreadsheet containing data values plotted in all main and supporting figures. (XLSX)

S1 Fig p53 is a target of CAPN3. **(A)** Both human p53 (hu-p53) and zebrafish p53 (zf-p53) contain two conserved putative CAPN3 recognition motifs, highlighted with colored shade. **(B)** Western blot of His-CAPN3 eluted by 20, 50, 100, 200, and 500 mM imidazole from the Ni-NTA agarose beads. His-CAPN3 was immunoblotted by a rabbit polyclonal antibody against CAPN3.
(TIF)

S2 Fig Nucleolar localisation of CAPN3 is Def-dependent. **(A)** Western blot of CAPN3^C129S^ and CAPN3^C129S-ΔNOLS^ in total protein extract (Total) and the nucleolar fraction (NO). 293T cells were transfected with respective plasmids and harvested at 72 h. **(B)** Western blot of hu-Def showing the knockdown of hu-Def by the *hu-def*–specific siRNAs. GAPDH: loading control. **(C)** Co-IP analysis of the interaction between HA-tagged Capn3b^C120S^ and Myc-tagged zebrafish Def, Def_S50, Def_S58,62A, or Def_S87,92A mutant proteins in 293T cells. Total protein was extracted from co-transfected cells at 72 h after transfection and was then incubated with Myc-beads. Western blot was performed with anti-HA and anti-Myc antibodies.
(TIF)

S3 Fig Expression and purification of (6xHis)-EGFP-D14-(6xHis) for mass spectrum analysis. (6xHis)-EGFP-D14-(6xHis) was expressed in *E*. *coli*. Total protein crude extract (CE) was treated with (CE+CIP @ 37°C) or without (CE @ 37°C) CIP at 37°C for 1 h. To purify (6xHis)-EGFP-D14-(6xHis), total protein crude extract was mixed with the Ni-NTA agarose beads followed by washing (wash 1) and eluting with 250 mM of imidazole (elution 1 and elution 2). Upper panel: Coomassie blue staining (CBB), lower panel: western blot of (6xHis)-EGFP-D14-(6xHis) using an antibody against zebrafish Def. Protein samples are as shown. FT, flow through.
(TIF)

S4 Fig Mass spectrum analysis of (6xHis)-EGFP-D14-(6xHis). **(A, B)** Mass spectra showing the precursor ions of the identified peptides bearing phosphorylated amino acid residues S50, S58, and S62 **(A)**, and S87 and S92 **(B)** in (6xHis)-EGFP-D14-(6xHis).
(TIF)

S5 Fig Alignment of the N-terminal amino acid sequences among zebrafish (1–189 aa), mouse (1–197 aa) and human (1–185 aa). The phosphorylated residues (S50, S58, S62, S87, and S92 in zebrafish Def) were highlighted.
(TIF)

S6 Fig Generation of transgenic fish lines. **(A)** Table listed the transgenic lines obtained. *Tg(fabp10a*:*def)* was obtained as described [20]. Two independent lines were obtained and used for each double mutant construct. Specific primer pair *def*_Fw1644 and *def*_Rv2048 was used in PCR to genotype transgenic fishes. **(B, C)** WISH analysis of *def* expression pattern in different transgenic fishes as shown using the *def* probe. The liver is highlighted with a red arrow.
(TIF)

S7 Fig Phosphorylation at the N-terminus does not affect Def nucleolar localisation. Co-immunostaining of Def or its mutant protein (in red) and nucleolar marker Fibrillarin (Fib, in green) in wild-type (WT), *def*^*-/-*^ mutant and different transgenic lines in the *def*^*-/-*^ background at 4 dpf as shown. DAPI was used to stain nuclei (in blue). Scale bar: 5 μm.
(TIF)

S8 Fig Liver development defect in *def*^*-/-*^*Tg(fabp10a*:*def_S58*,*62A)* and *def*^*-/-*^*Tg(fabp10a*:*def_S87*,*92A)* is not due to cell apoptosis. **(A)** Representative images showing TUNEL assay for detecting the apoptotic cells in the liver and other organs of 4-dpf embryos in different genotypes as indicated. White arrows indicate the apoptotic cells observed in the epidermis and neural tube. Asterisk indicates the site of the embryonic liver. WT embryo section pretreated with DNase I (WT+DNase I) was used as the positive control. Scale bar: 50 μm. **(B)** Summary of TUNEL assay in different genotypes.
(TIF)

S9 Fig Classification of PCNA positive cells. **(A–E)** 3-dpf WT embryo sections were co-stained with an anti-PCNA antibody, anti-Fabp10a antibody (to show hepatocytes), and DAPI (to stain nuclei). The high magnification of the region outlined by white box in **(A)** is presented to show PCNA signal **(B)**, Fabp10a signal **(C)**, DAPI staining **(D)**, and merged image **(E)**. Hepatocytes harboring distinct brilliant foci (highlighted with a white arrow in **B**) were considered to be cells at S-phase, whereas cells with even distributed PCNA signal to be at non-S-phase. Scale bar: 5 μm.
(TIF)

S10 Fig Simultaneous phosphorylations at S58 and S62 or at S87 and S92 are necessary for Def to promote cell cycle progression. **(A–D)** A representative image showing the liver bud in a *Tg(fabp10a*:*dsRed; elastase*:*GFP)* reporter fish **(A)** and a dissected liver bud from the same reporter fish (two insets in **A**: left, showing the DsRed-labelled liver bud; right, a bright field image of the liver bud). Each transgenic fish line was crossed to the *Tg(fabp10a*:*dsRed; elastase*:*GFP)* background and raised to 8 dpf. Livers of different genotyped fish were dissected under a fluorescent microscope, and cells were dissociated. Dissociated cells were co-stained with DAPI (to stain nuclei) **(B)** and the DsRed fluorescence **(C)** for counting the number of DsRed-positive cells (hepatocytes) **(D)**. In each case, more than 94% of the cells were found to be DsRed-positive cells. Scale bar: 100 μm. **(E)** Flow cytometry analysis of the liver cells isolated from 8-dpf zebrafish of different genotypes as indicated. Representative graph from three independent experiments is shown here. Statistical analysis of the flow cytometry data is shown in Fig 8A. Underlying data for **(E)** are provided in S1 Data.
(TIF)

S11 Fig Knockdown of CAPN3 or hu-Def causes a p53-dpendent cell cycle arrest. Graph showing the flow cytometry analysis of the HCT116-p53^+/+^ or HCT116-p53^-/-^ cells at 24 h after treatment with ctrl-siRNA, *capn3*-siRNA, or *hu-def*-siRNA. Representative graph from three independent experiments is shown. Statistical analysis of the flow cytometry data is shown in Fig 8C. Underlying data are provided in S1 Data.
(TIF)

S12 Fig Knockdown of p53 can partially rescue the cell cycle arrest in *def*^*-/-*^. **(A)** Western blot of p53 showing the knockdown of p53 by *p53*-specific morpholino mixes (ATG-MO plus spl-MO) in zebrafish embryos at 3 dpf. β-actin: loading control. **(B)** Representative images of P-H3 and PCNA immunostaining in 3-dpf old *def*^*-/-*^ embryos injected with *p53*-specific morpholino mixes (ATG-MO plus spl-MO). Scale bar: 100 μm. Hepatocytes are marked by the Bhmt antibody (images for P-H3 staining) or Fabp10a antibody (images for PCNA staining). Nuclei are stained by DAPI. Liver area is outlined by a dashed line.
(TIF)

S13 Fig p53^R175H^ is one of the hotspots for cancer mutation in humans. **(A)** Western blot analysis of p53, Def, Fib, and NPC in the cytoplasm (CP), nucleoplasm (NP), and nucleolar (NO) fractions extracted from three different breast cancer cell lines: MCF-7 (p53^WT^), SK-BR-3 (p53^R175H^), and JIMTI (p53^R248W^). The basal levels of p53 in these cell lines were very different. **(B)** Histogram showing the p53 NO/NP ratios in three different cancer cells by grey-value analysis of the band intensity shown in **(A)**. **(C)** p53^R175^ is one of the hotspots for cancer mutation, with 1,260 tumour samples harbouring the R175 substitution mutation. Scale bar on the top represents the amino acid position in p53. **(D)** p53^R175H^ mutation ranks first among the p53 substitution mutations in cancer samples. **(E)** p53^R175H^ is not specific for any cancer type when compared with other p53 mutations A138V, M237I, and R248W. All four of these p53 mutations were observed more frequently in large intestine, breast, haematopoietic and lymphoid, and ovary tumour samples. The *y*-axis indicates the percentage of tumour of a specific tissue in total tumour samples of the p53 A138V, R175H, M237I, or R248W substitution recorded. Data shown in **(C–E)** were retrieved from the Catalogue of Somatic Mutations in Cancer database (http://cancer.sanger.ac.uk/cosmic). Underlying data for **(B)**, **(C)**, and **(E)** are provided in S1 Data.
(TIF)

S1 Table Number of embryos used and cells counted for nucleolar Capn3b-positive cells in different genotypes. (DOCX)

S2 Table Counting P-H3-, EdU-, and PCNA-positive hepatocytes in different genotypes. (DOCX)

S3 Table Number of embryos and sections used in counting P-H3-, EdU-, and PCNA-positive cells in different genotypes. (DOCX)

S4 Table Counting P-H3-positive hepatocytes in different genotypes. (DOCX)

S5 Table Number of embryos, sections, and cells used in measuring p53 intensity in different genotypes. (DOCX)

S6 Table PCR primers used for site‐direct mutagenesis for *EGFP-D14*-pCS2^+^ (mutated bases in lower case). (DOCX)

S7 Table PCR primers used for site‐direct mutagenesis for *myc*-*def*-pCS2^+^ (mutated bases in lower case). (DOCX)

S8 Table PCR primers used for site‐direct mutagenesis for *myc*-*p53-HA*-pCDNA^+^ (mutated bases in lower case). (DOCX)

S9 Table PCR primers used for site‐direct mutagenesis for *CAPN3*-pCDNA^+^ or *CAPN3a/b*-pCS2+(mutated bases in lower case). (DOCX)

## Abbreviations

CAPN3: calpain-3
CBB: Coomassie brilliant blue
CIP: calf intestinal alkaline phosphatase
Co-IP: co-immunoprecipitation
CP: cytoplasm
DBD: DNA-binding domain
Def: Digestive organ expansion factor
dpf: days post fertilisation
EDTA: ethylene diaminetetraacetic acid
EGFP: enhanced green fluorescent protein
Fib: Fibrillarin
hpf: hours post fertilisation
hu-Def: human Def
MO: morpholino
MS: mass spectrometry
NO: nucleoli
NOLS: nucleolar localisation signal
NP: nucleoplasm
NPC: nuclear pore complex
P-H3: phosphorylated histone 3
SEM: standard error of the mean
SSU: small subunit
TUNEL: terminal deoxynucleotidyl transferase dUTP nick-end labelling
WISH: whole-mount in situ hybridisation
