# Supplementary figures and images for: Phosphorylation of Def Regulates Nucleolar p53 Turnover and Cell Cycle Progression through Def Recruitment of Calpain3
Source: PLoS Biol. 2016 Sep 22;14(9):e1002555. doi: 10.1371/journal.pbio.1002555 (PMC5033581; doi:10.1371/journal.pbio.1002555)

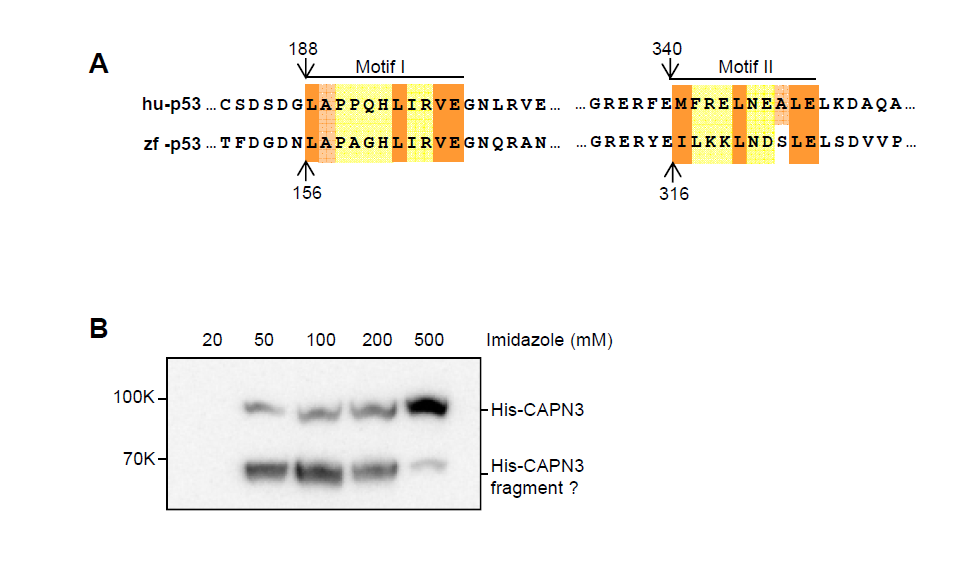

Supplement: S1 Fig — (A) Both human p53 (hu-p53) and zebrafish p53 (zf-p53) contain two conserved putative CAPN3 recognition motifs, highlighted with colored shade. (B) Western blot of His-CAPN3 eluted by 20, 50, 100, 200, and 500 mM imidazole from the Ni-NTA agarose beads. His-CAPN3 was immunoblotted by a rabbit polyclonal antibody against CAPN3. (TIF) [file pbio.1002555.s002.tif]

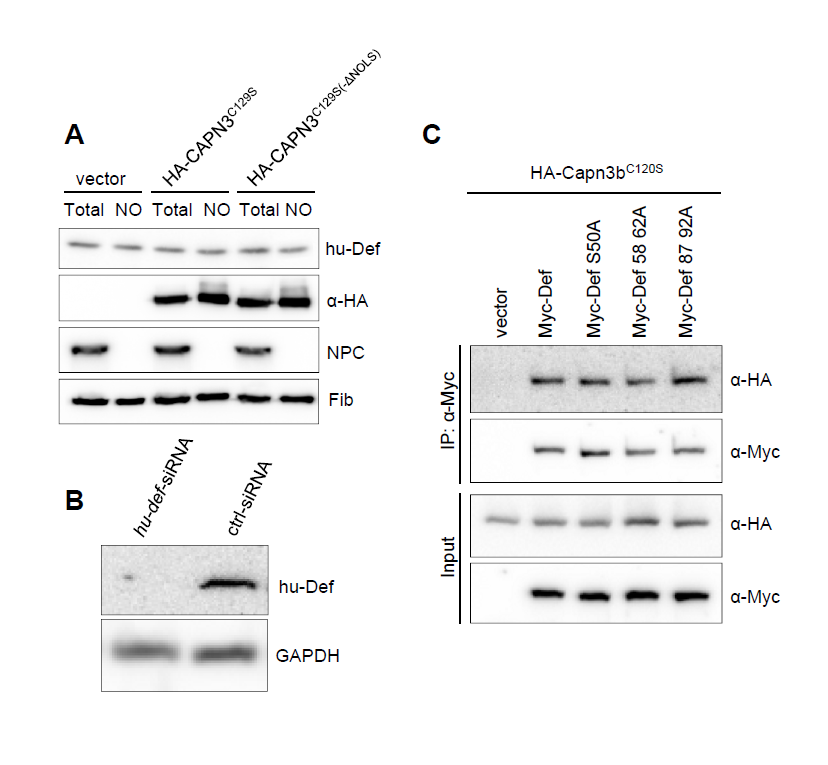

Supplement: S2 Fig — (A) Western blot of CAPN3C129S and CAPN3C129S-ΔNOLS in total protein extract (Total) and the nucleolar fraction (NO). 293T cells were transfected with respective plasmids and harvested at 72 h. (B) Western blot of hu-Def showing the knockdown of hu-Def by the hu-def–specific siRNAs. GAPDH: loading control. (C) Co-IP analysis of the interaction between HA-tagged Capn3bC120S and Myc-tagged zebrafish Def, Def_S50, Def_S58,62A, or Def_S87,92A mutant proteins in 293T cells. Total protein was extracted from co-transfected cells at 72 h after transfection and was then incubated with Myc-beads. Western blot was performed with anti-HA and anti-Myc antibodies. (TIF) [file pbio.1002555.s003.tif]

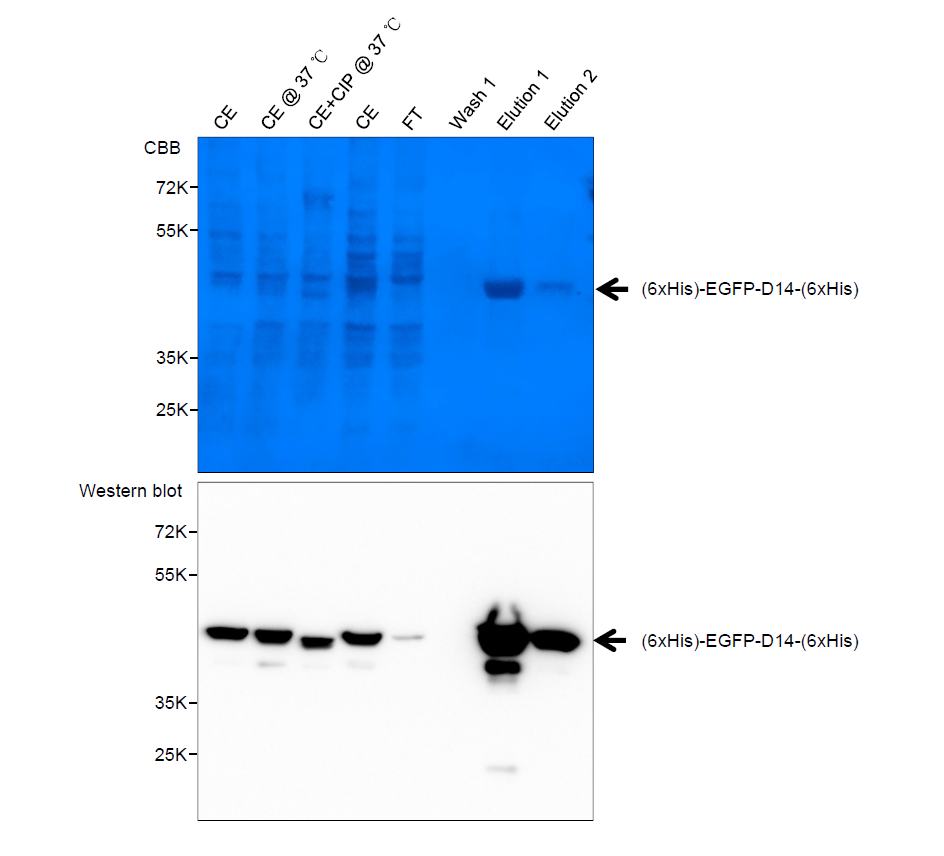

Supplement: S3 Fig — (6xHis)-EGFP-D14-(6xHis) was expressed in E. coli. Total protein crude extract (CE) was treated with (CE+CIP @ 37°C) or without (CE @ 37°C) CIP at 37°C for 1 h. To purify (6xHis)-EGFP-D14-(6xHis), total protein crude extract was mixed with the Ni-NTA agarose beads followed by washing (wash 1) and eluting with 250 mM of imidazole (elution 1 and elution 2). Upper panel: Coomassie blue staining (CBB), lower panel: western blot of (6xHis)-EGFP-D14-(6xHis) using an antibody against zebrafish Def. Protein samples are as shown. FT, flow through. (TIF) [file pbio.1002555.s004.tif]

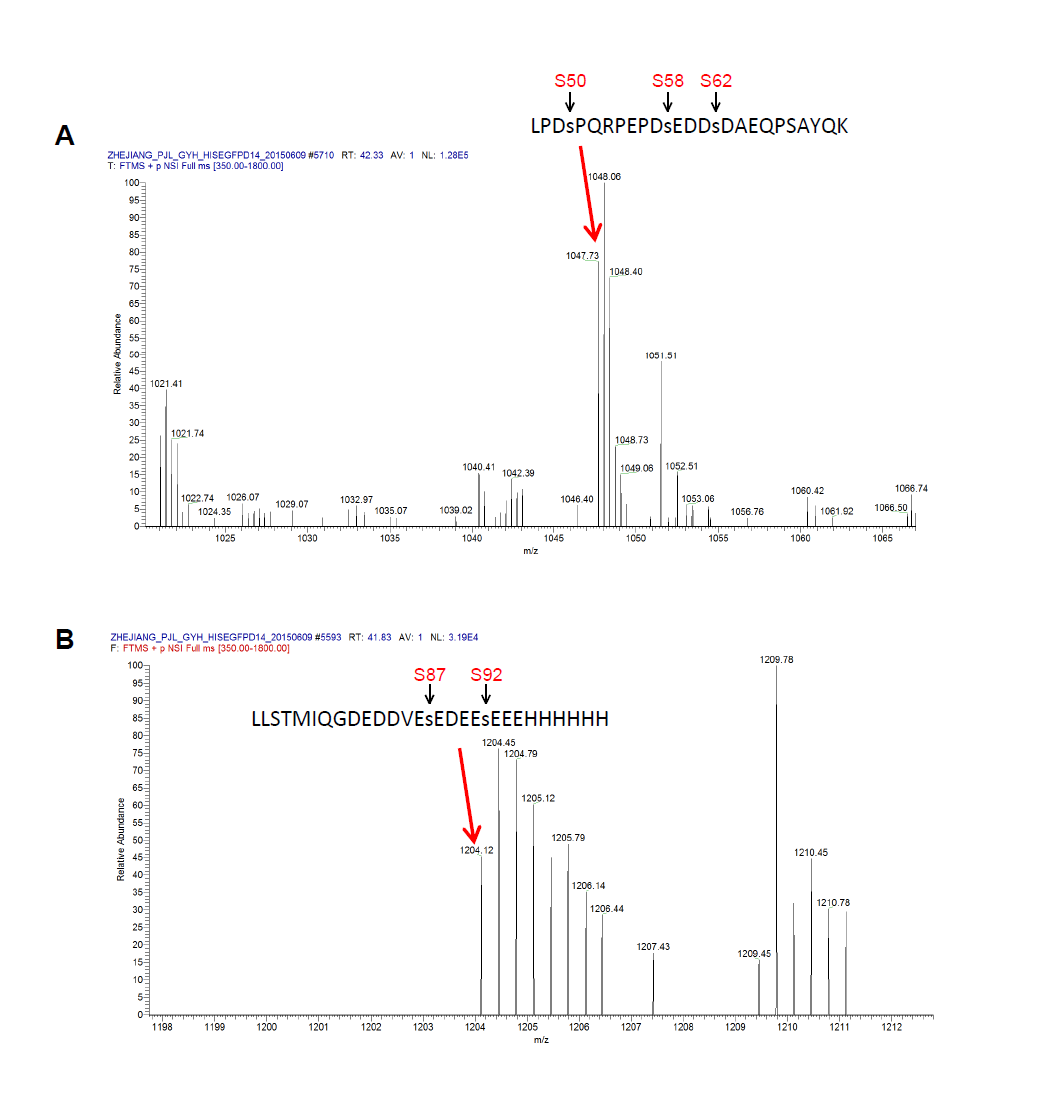

Supplement: S4 Fig — (A, B) Mass spectra showing the precursor ions of the identified peptides bearing phosphorylated amino acid residues S50, S58, and S62 (A), and S87 and S92 (B) in (6xHis)-EGFP-D14-(6xHis). (TIF) [file pbio.1002555.s005.tif]

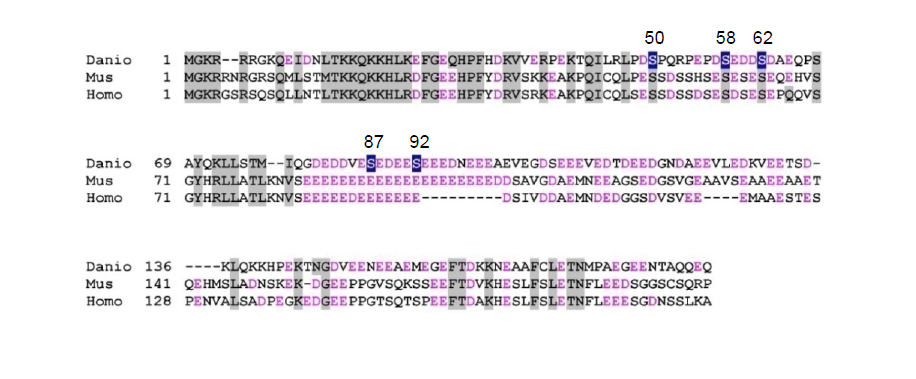

Supplement: S5 Fig — The phosphorylated residues (S50, S58, S62, S87, and S92 in zebrafish Def) were highlighted. (TIF) [file pbio.1002555.s006.tif]

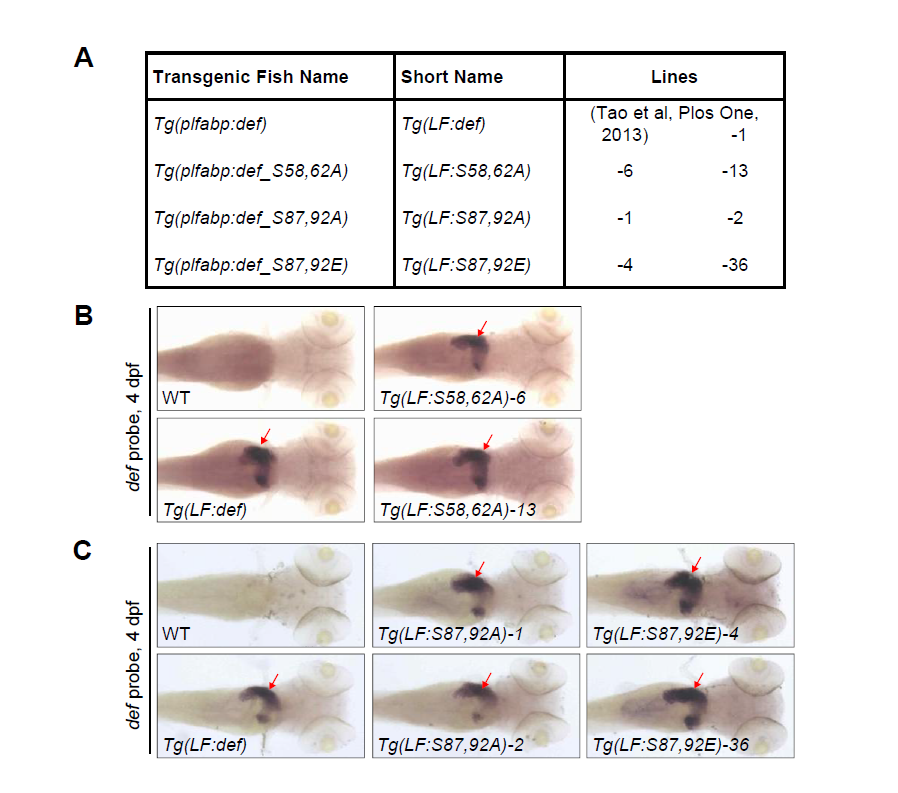

Supplement: S6 Fig — (A) Table listed the transgenic lines obtained. Tg(fabp10a:def) was obtained as described [20]. Two independent lines were obtained and used for each double mutant construct. Specific primer pair def_Fw1644 and def_Rv2048 was used in PCR to genotype transgenic fishes. (B, C) WISH analysis of def expression pattern in different transgenic fishes as shown using the def probe. The liver is highlighted with a red arrow. (TIF) [file pbio.1002555.s007.tif]

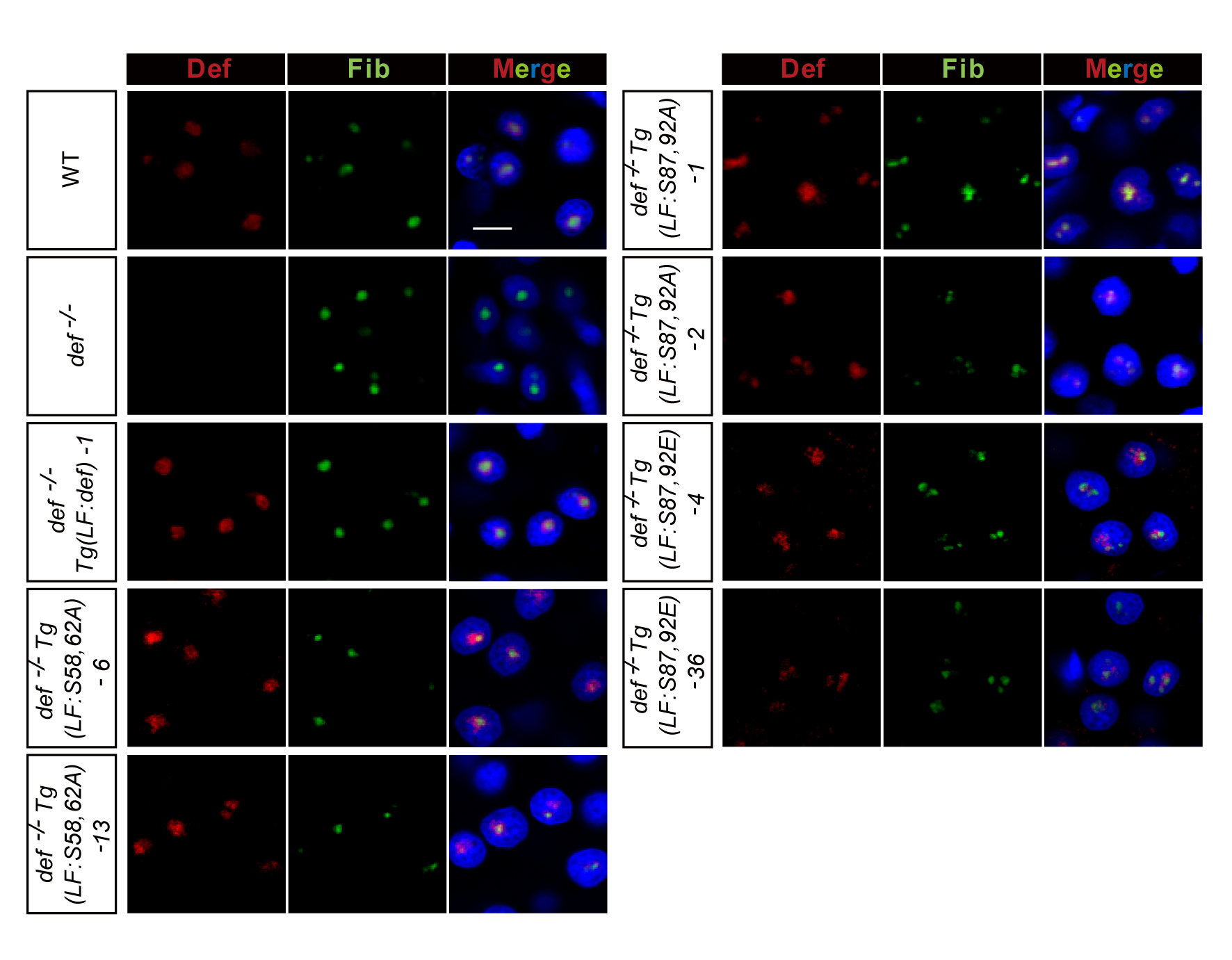

Supplement: S7 Fig — Co-immunostaining of Def or its mutant protein (in red) and nucleolar marker Fibrillarin (Fib, in green) in wild-type (WT), def-/- mutant and different transgenic lines in the def-/- background at 4 dpf as shown. DAPI was used to stain nuclei (in blue). Scale bar: 5 μm. (TIF) [file pbio.1002555.s008.tif]

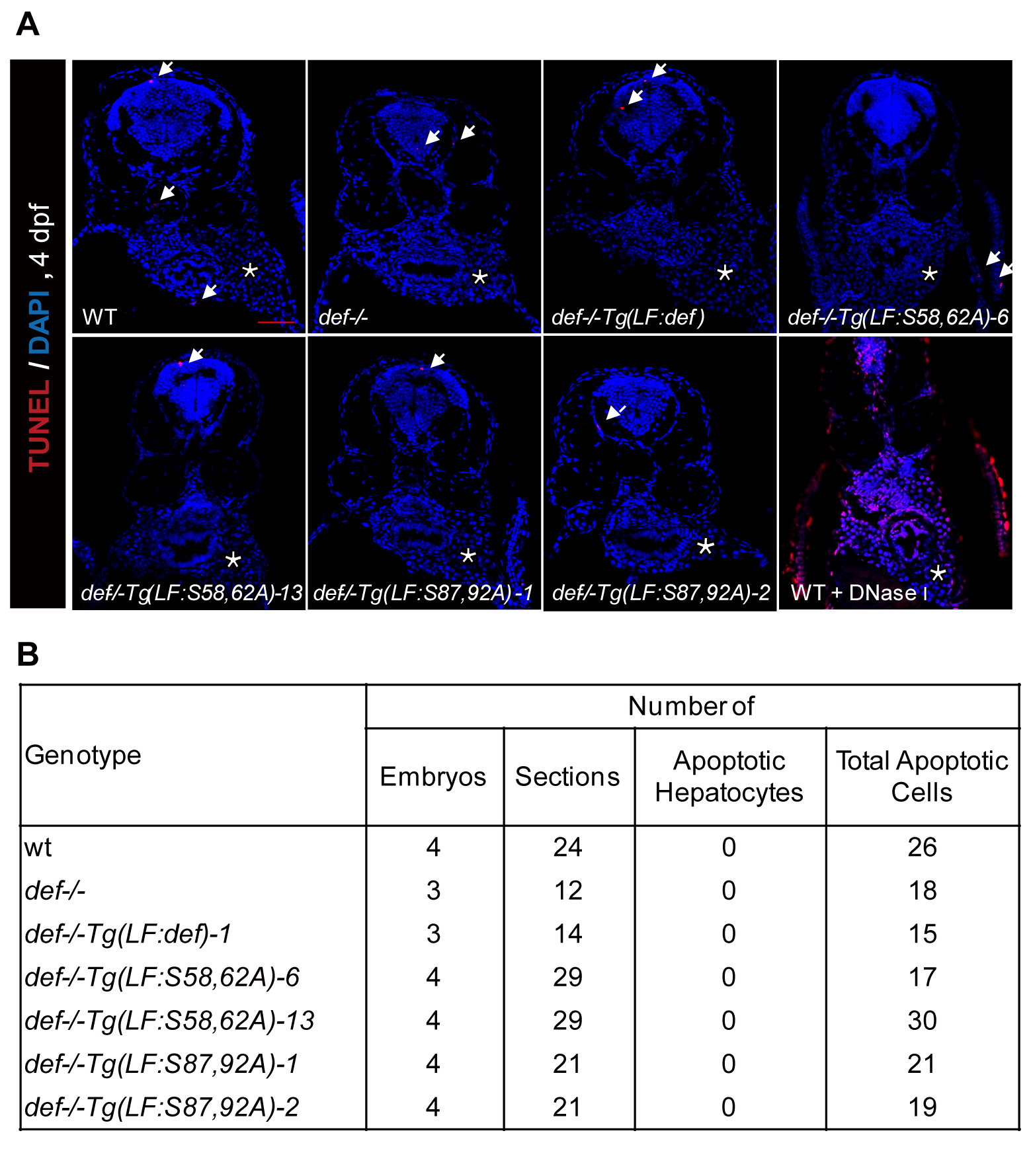

Supplement: S8 Fig — (A) Representative images showing TUNEL assay for detecting the apoptotic cells in the liver and other organs of 4-dpf embryos in different genotypes as indicated. White arrows indicate the apoptotic cells observed in the epidermis and neural tube. Asterisk indicates the site of the embryonic liver. WT embryo section pretreated with DNase I (WT+DNase I) was used as the positive control. Scale bar: 50 μm. (B) Summary of TUNEL assay in different genotypes. (TIF) [file pbio.1002555.s009.tif]

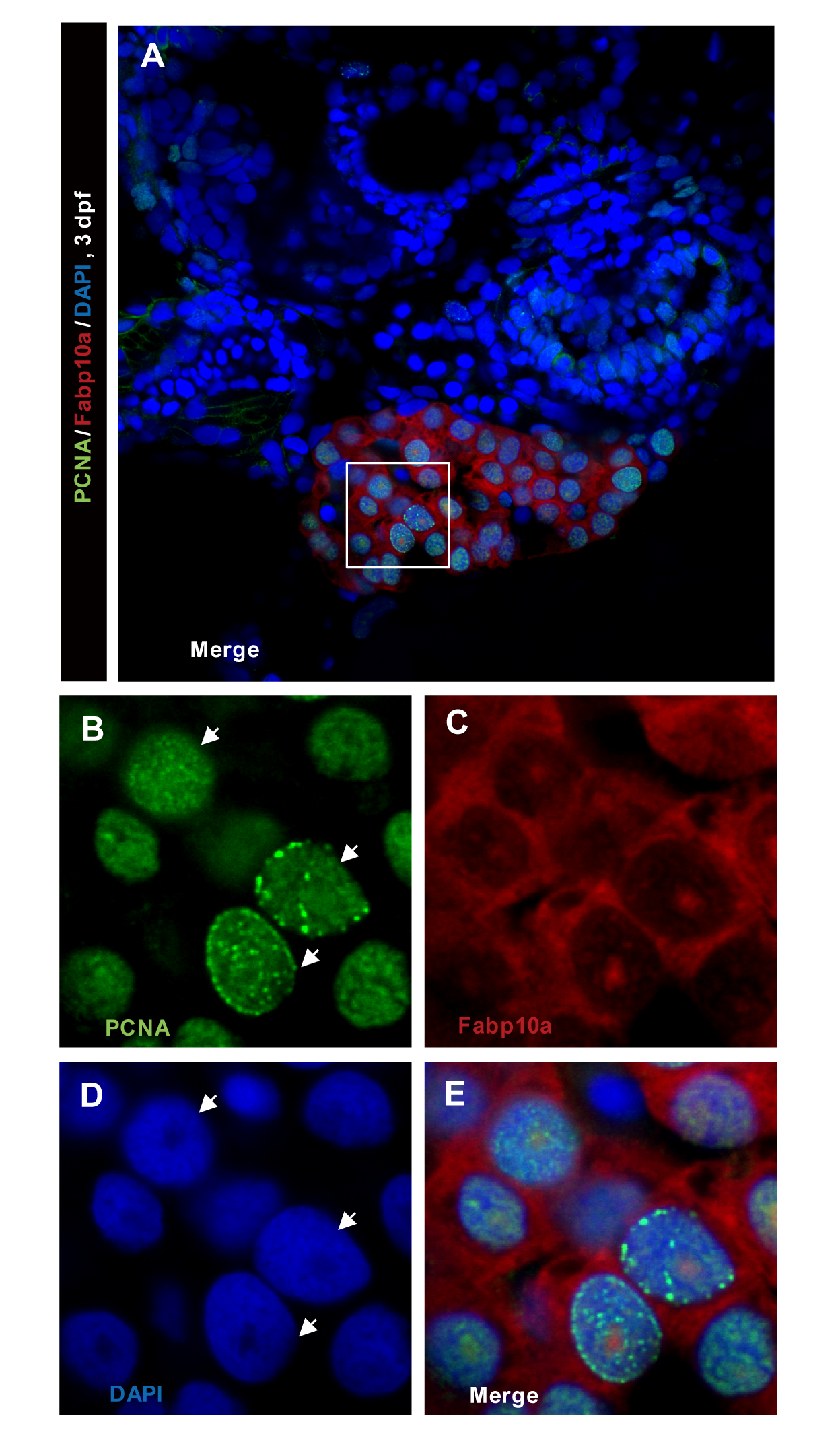

Supplement: S9 Fig — (A–E) 3-dpf WT embryo sections were co-stained with an anti-PCNA antibody, anti-Fabp10a antibody (to show hepatocytes), and DAPI (to stain nuclei). The high magnification of the region outlined by white box in (A) is presented to show PCNA signal (B), Fabp10a signal (C), DAPI staining (D), and merged image (E). Hepatocytes harboring distinct brilliant foci (highlighted with a white arrow in B) were considered to be cells at S-phase, whereas cells with even distributed PCNA signal to be at non-S-phase. Scale bar: 5 μm. (TIF) [file pbio.1002555.s010.tif]

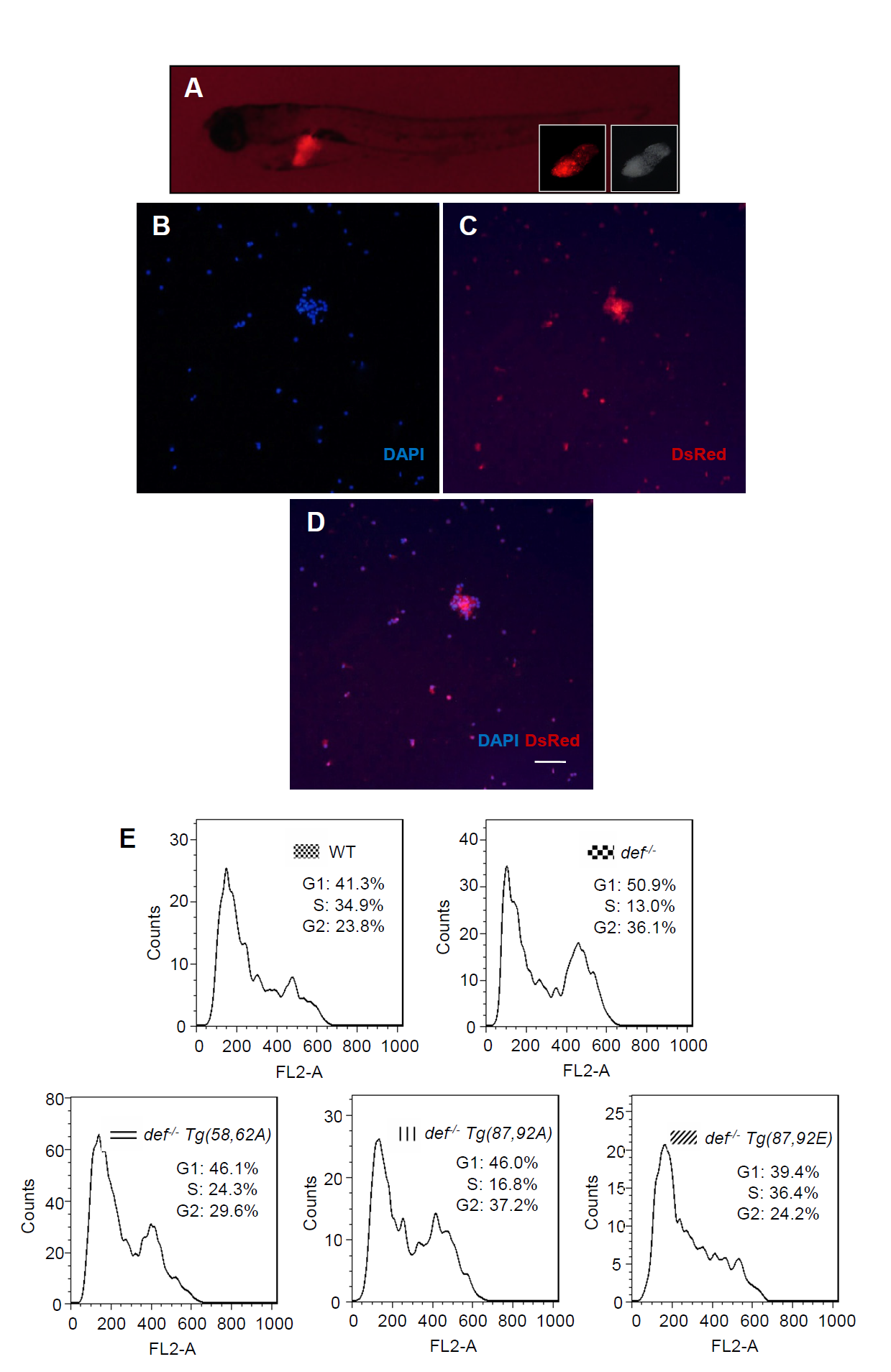

Supplement: S10 Fig — (A–D) A representative image showing the liver bud in a Tg(fabp10a:dsRed; elastase:GFP) reporter fish (A) and a dissected liver bud from the same reporter fish (two insets in A: left, showing the DsRed-labelled liver bud; right, a bright field image of the liver bud). Each transgenic fish line was crossed to the Tg(fabp10a:dsRed; elastase:GFP) background and raised to 8 dpf. Livers of different genotyped fish were dissected under a fluorescent microscope, and cells were dissociated. Dissociated cells were co-stained with DAPI (to stain nuclei) (B) and the DsRed fluorescence (C) for counting the number of DsRed-positive cells (hepatocytes) (D). In each case, more than 94% of the cells were found to be DsRed-positive cells. Scale bar: 100 μm. (E) Flow cytometry analysis of the liver cells isolated from 8-dpf zebrafish of different genotypes as indicated. Representative graph from three independent experiments is shown here. Statistical analysis of the flow cytometry data is shown in Fig 8A. Underlying data for (E) are provided in S1 Data. (TIF) [file pbio.1002555.s011.tif]

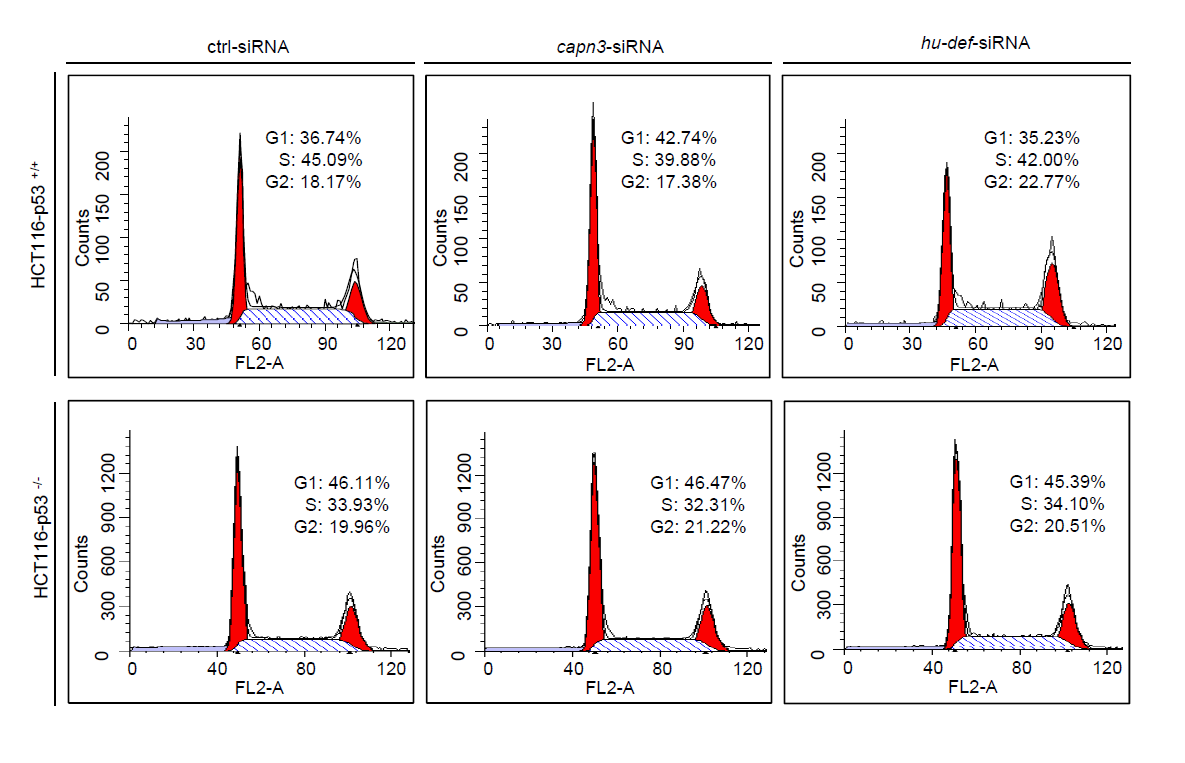

Supplement: S11 Fig — Graph showing the flow cytometry analysis of the HCT116-p53+/+ or HCT116-p53-/- cells at 24 h after treatment with ctrl-siRNA, capn3-siRNA, or hu-def-siRNA. Representative graph from three independent experiments is shown. Statistical analysis of the flow cytometry data is shown in Fig 8C. Underlying data are provided in S1 Data. (TIF) [file pbio.1002555.s012.tif]

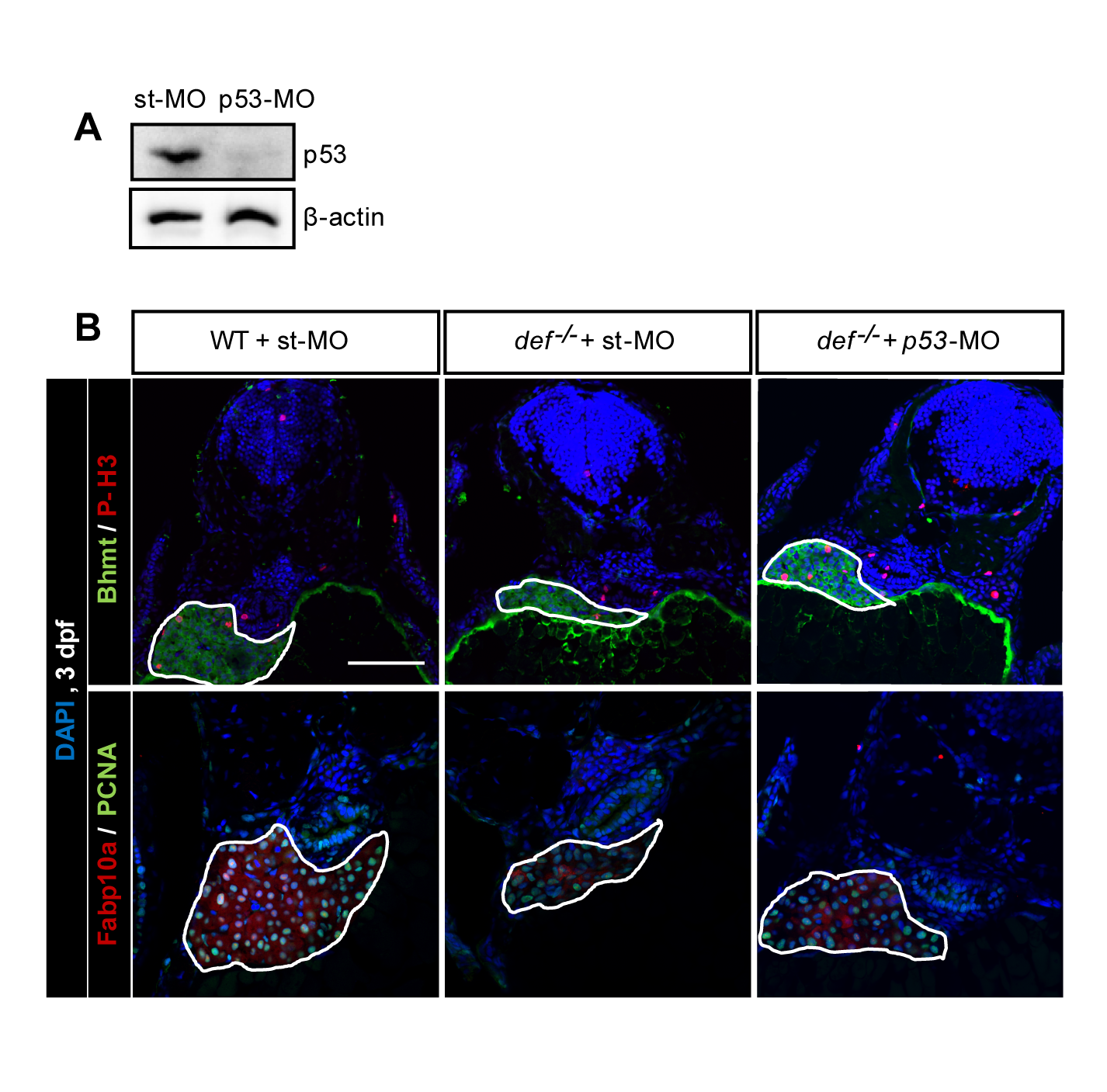

Supplement: S12 Fig — (A) Western blot of p53 showing the knockdown of p53 by p53-specific morpholino mixes (ATG-MO plus spl-MO) in zebrafish embryos at 3 dpf. β-actin: loading control. (B) Representative images of P-H3 and PCNA immunostaining in 3-dpf old def-/- embryos injected with p53-specific morpholino mixes (ATG-MO plus spl-MO). Scale bar: 100 μm. Hepatocytes are marked by the Bhmt antibody (images for P-H3 staining) or Fabp10a antibody (images for PCNA staining). Nuclei are stained by DAPI. Liver area is outlined by a dashed line. (TIF) [file pbio.1002555.s013.tif]

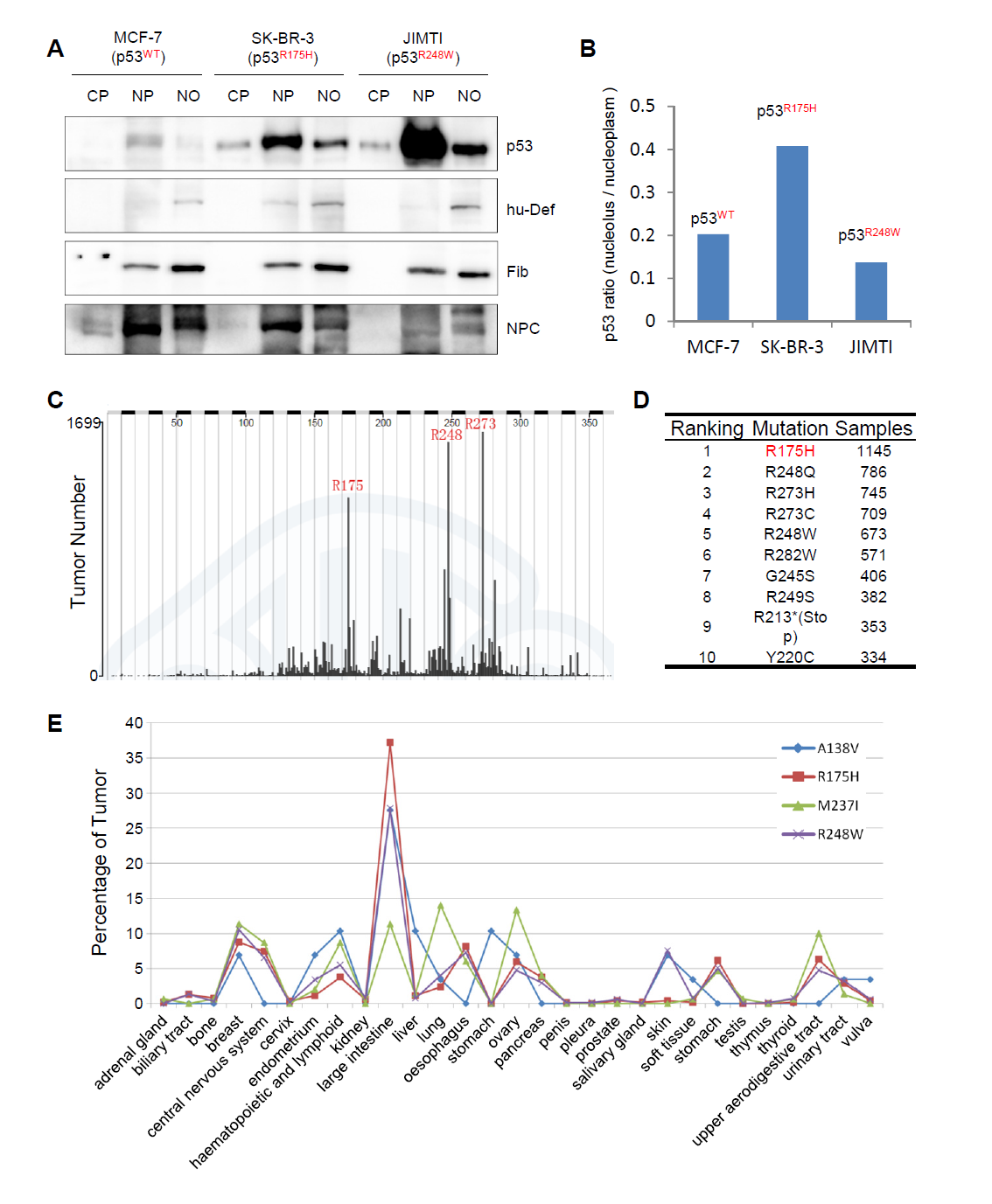

Supplement: S13 Fig — (A) Western blot analysis of p53, Def, Fib, and NPC in the cytoplasm (CP), nucleoplasm (NP), and nucleolar (NO) fractions extracted from three different breast cancer cell lines: MCF-7 (p53WT), SK-BR-3 (p53R175H), and JIMTI (p53R248W). The basal levels of p53 in these cell lines were very different. (B) Histogram showing the p53 NO/NP ratios in three different cancer cells by grey-value analysis of the band intensity shown in (A). (C) p53R175 is one of the hotspots for cancer mutation, with 1,260 tumour samples harbouring the R175 substitution mutation. Scale bar on the top represents the amino acid position in p53. (D) p53R175H mutation ranks first among the p53 substitution mutations in cancer samples. (E) p53R175H is not specific for any cancer type when compared with other p53 mutations A138V, M237I, and R248W. All four of these p53 mutations were observed more frequently in large intestine, breast, haematopoietic and lymphoid, and ovary tumour samples. The y-axis indicates the percentage of tumour of a specific tissue in total tumour samples of the p53 A138V, R175H, M237I, or R248W substitution recorded. Data shown in (C–E) were retrieved from the Catalogue of Somatic Mutations in Cancer database (http://cancer.sanger.ac.uk/cosmic). Underlying data for (B), (C), and (E) are provided in S1 Data. (TIF) [file pbio.1002555.s014.tif]
