# Supplementary material for: Phosphorylation of Def Regulates Nucleolar p53 Turnover and Cell Cycle Progression through Def Recruitment of Calpain3
Source: PLoS Biol. 2016 Sep 22;14(9):e1002555. doi: 10.1371/journal.pbio.1002555 (PMC5033581; doi:10.1371/journal.pbio.1002555)
Supplement: S1 Table — (DOCX) [file pbio.1002555.s015.docx]

| **S1 Table** | | |
| --- | --- | --- |
| Genotype | Embryos | Counted Cells |
| WT | 3 | 144 |
| *def-/-* | 3 | 97 |
| *def-/-Tg(LF:def)-1* | 3 | 101 |
| *def-/-Tg(LF:S87,92A)-1* | 3 | 195 |
| *def-/-Tg(LF:S87,92E)-4* | 3 | 212 |
