# Supplementary material for: Phosphorylation of Def Regulates Nucleolar p53 Turnover and Cell Cycle Progression through Def Recruitment of Calpain3
Source: PLoS Biol. 2016 Sep 22;14(9):e1002555. doi: 10.1371/journal.pbio.1002555 (PMC5033581; doi:10.1371/journal.pbio.1002555)
Supplement: S2 Table — (DOCX) [file pbio.1002555.s016.docx]

| **S2 Table** | | | | | | |
| --- | --- | --- | --- | --- | --- | --- |
| Genotype | Positive Cells Number / Counted Hepatocytes Total Number | | | | | |
|  | P-H3 | | EdU | | PCNA | |
|  | 2.5 dpf | 3 dpf | 2.5 dpf | 3 dpf | 2.5 dpf | 3 dpf |
| wt | 122 / 1862 | 193 / 3733 | 1296 / 2544 | 1176 / 3377 | 605 / 1799 | 997 / 3913 |
| *def-/-* | 45 / 1507 | 55 / 2212 | 380 / 1194 | 275 / 2060 | 89 / 863 | 103 / 1006 |
| *def-/-Tg(LF:def)* | 90 / 1265 | 115 / 2107 | 968 / 1778 | 936 / 2648 | 228 / 756 | 524 / 1929 |
| *def-/-Tg(LF:S58,62A)-6* | 60 / 1498 | 104 / 2899 | 841 / 1942 | 1027 / 4154 | 184 / 885 | 364 / 2104 |
| *def-/-Tg(LF:S58,62A)-13* | 104 / 2302 | 160 / 4413 | 1119 / 2690 | 846 / 3007 | 446 / 2019 | 488 / 2721 |
