# Supplementary material for: Phosphorylation of Def Regulates Nucleolar p53 Turnover and Cell Cycle Progression through Def Recruitment of Calpain3
Source: PLoS Biol. 2016 Sep 22;14(9):e1002555. doi: 10.1371/journal.pbio.1002555 (PMC5033581; doi:10.1371/journal.pbio.1002555)
Supplement: S3 Table — (DOCX) [file pbio.1002555.s017.docx]

| **S3 Table** | | | | | | |
| --- | --- | --- | --- | --- | --- | --- |
| Genotype | Number of embryos / sections | | | | | |
|  | P-H3 | | EdU | | PCNA | |
|  | 2.5 dpf | 3 dpf | 2.5 dpf | 3 dpf | 2.5 dpf | 3 dpf |
| wt | 6 / 49 | 6 / 61 | 6 / 39 | 6 / 56 | 7 / 52 | 7 / 61 |
| *def-/-* | 6 / 40 | 6 / 46 | 6 / 35 | 6 / 38 | 6 / 46 | 6 / 33 |
| *def-/-Tg(LF:def)* | 5 / 44 | 5 / 47 | 6 / 43 | 6 / 44 | 6 / 39 | 6 / 36 |
| *def-/-Tg(LF:S58,62A)-6* | 5 / 44 | 5 / 46 | 6 / 39 | 6 / 55 | 6 / 40 | 6 / 47 |
| *def-/-Tg(LF:S58,62A)-13* | 6 / 50 | 6 / 58 | 7 / 52 | 5 / 38 | 7 / 58 | 7 / 56 |
