# Supplementary material for: Phosphorylation of Def Regulates Nucleolar p53 Turnover and Cell Cycle Progression through Def Recruitment of Calpain3
Source: PLoS Biol. 2016 Sep 22;14(9):e1002555. doi: 10.1371/journal.pbio.1002555 (PMC5033581; doi:10.1371/journal.pbio.1002555)
Supplement: S4 Table — (DOCX) [file pbio.1002555.s018.docx]

| **S4 Table** | | | | | | | | |
| --- | --- | --- | --- | --- | --- | --- | --- | --- |
| Genotype | Number of | | | | | | | |
|  | 2.5 dpf | | | | 3 dpf | | | |
|  | Embryos | Sections | Positive Cells | Total Counted Cells | Embryos | Sections | Positive Cells | Total  Counted  Cells |
| wt | 5 | 41 | 108 | 1658 | 5 | 46 | 153 | 3498 |
| *def-/-* | 5 | 30 | 28 | 895 | 5 | 39 | 40 | 1704 |
| *def-/-Tg(LF:def)* | 5 | 44 | 57 | 967 | 5 | 47 | 126 | 2884 |
| *def-/-Tg(LF:S87,92A)-1* | 5 | 27 | 43 | 1023 | 6 | 36 | 65 | 2054 |
| *def-/-Tg(LF:S87,92A)-2* | 5 | 28 | 38 | 862 | 6 | 46 | 96 | 3168 |
