# Supplementary material for: Phosphorylation of Def Regulates Nucleolar p53 Turnover and Cell Cycle Progression through Def Recruitment of Calpain3
Source: PLoS Biol. 2016 Sep 22;14(9):e1002555. doi: 10.1371/journal.pbio.1002555 (PMC5033581; doi:10.1371/journal.pbio.1002555)
Supplement: S5 Table — (DOCX) [file pbio.1002555.s019.docx]

| **S5 Table** | | | | | | |
| --- | --- | --- | --- | --- | --- | --- |
| Genotype | Number of | | | | | |
|  | Liver | | | Gut | | |
|  | Embryos | Sections | Counted Cells* | Embryos | Sections | Counted Cells* |
| WT | 3  3  3  3  3  3 | 10  9  9  9  10  9 | 150  135  135  135  150  135 | 3  3  3  3  3  3 | 9  9  9  9  9  9 | 135  135  135  135  135  135 |
| *def-/-* |  |  |  |  |  |  |
| *def-/-Tg(LF:def)* |  |  |  |  |  |  |
| *def-/-Tg(LF:S58,62A)-6* |  |  |  |  |  |  |
| *def-/-Tg(LF:S58,62A)-13*  *def-/-Tg(LF:S87,92A)-1* |  |  |  |  |  |  |
| *def-/-Tg(LF:S87,92A)-2*  *def-/-Tg(LF:S87,92E)-4*  *def-/-Tg(LF:S87,92E)-36* | 3  3  3 | 11  9  9 | 165  135  135 | 3  3  3 | 9  9  9 | 135  135  135 |
| * 15 cells in each section were randomly picked for p53 intensity measurement. | | | | | | |
