# Supplementary material for: Phosphorylation of Def Regulates Nucleolar p53 Turnover and Cell Cycle Progression through Def Recruitment of Calpain3
Source: PLoS Biol. 2016 Sep 22;14(9):e1002555. doi: 10.1371/journal.pbio.1002555 (PMC5033581; doi:10.1371/journal.pbio.1002555)
Supplement: S6 Table — (DOCX) [file pbio.1002555.s020.docx]

| **S6 Table** | | | |
| --- | --- | --- | --- |
| **Construct** | | **Forward primer (5’-3’) (Fw)** | **Reverse primer (5’-3’) (Rv)** |
| *T15A* | ATTGATAATTTAgCTAAGAAACAGAAGAAACATCTAAAGGAATTTGGTGA | | TCTGTTTCTTAGcTAAATTATCAATCTCCTGTTTTCCTCGCCTTCTTTTG |
| *S50A* | CGCTTGCCTGACgcTCCACAGCGCCCTGAACCAGACAGTGAGGATGACAG | | GGGCGCTGTGGAgcGTCAGGCAAGCGCAAAATCTGAGTCTTCTCCGGTCT |
| *S58A* | CCTGAACCAGACgcTGAGGATGACAGTGATGCTGAGCAACCATCTGCATA | | CTGTCATCCTCAgcGTCTGGTTCAGGGCGCTGTGGACTGTCAGGCAAGCG |
| *S62A* | AGTGAGGATGACgcTGATGCTGAGCAACCATCTGCATATCAGAAACTACT | | TGCTCAGCATCAgcGTCATCCTCACTGTCTGGTTCAGGGCGCTGTGGACT |
| *S68A* | GCTGAGCAACCAgCTGCATATCAGAAACTACTGTCCACCATGATTCAAGG | | TCTGATATGCAGcTGGTTGCTCAGCATCACTGTCATCCTCACTGTCTGGT |
| *Y70A* | CAACCATCTGCAgcTCAGAAACTACTGTCCACCATGATTCAAGGTGATGA | | AGTAGTTTCTGAgcTGCAGATGGTTGCTCAGCATCACTGTCATCCTCACT |
| *T76A* | AAACTACTGTCCgCCATGATTCAAGGTGATGAAGATGATGTTGAGAGTGA | | CTTGAATCATGGcGGACAGTAGTTTCTGATATGCAGATGGTTGCTCAGCA |
| *T42A* | AGACCGGAGAAGgCTCAGATTTTGCGCTTGCCTGACAGTCCACAGCGCC | | GCAAAATCTGAGcCTTCTCCGGTCTTTCAACAACCTTATCGTGAAAAGG |
| *S75A* | CAGAAACTACTGgCCACCATGATTCAAGGTGATGAAGATGATGTTGAGA | | GAATCATGGTGGcCAGTAGTTTCTGATATGCAGATGGTTGCTCAGCATC |
| *S87A* | GATGATGTTGAGgcTGAAGATGAAGAAAGCGAGGAAGAGTGAGAATTCAA | | TCTTCATCTTCAgcCTCAACATCATCTTCATCACCTTGAATCATGGTGGA |
| *S92A* | GAAGATGAAGAAgcCGAGGAAGAGTGAGAATTCAAGGCCTCTCGAGCCT | | CACTCTTCCTCGgcTTCTTCATCTTCACTCTCAACATCATCTTCATCAC |
| *S50,58A* | as “*S58A”* based on “*S50A”* | | CTGTCATCCTCAgcGTCTGGTTCAGGGCGCTGTGGAgcGTCAGGCAAGCG |
| *S50,62A* | as “*S62A”* based on *“S50A”* | | TGCTCAGCATCAgcGTCATCCTCACTGTCTGGTTCAGGGCGCTGTGGAgc |
| *S58,62A* | CCAGACgcTGAGGATGACgcTGATGCTGAGCAACCATCTGCATATCAGAA | | GCATCAgcGTCATCCTCAgcGTCTGGTTCAGGGCGCTGTGGACTGTCAGG |
| *S50,58,62A* | as “*S58,62A”* based on *“S50A”* | | GCATCAgcGTCATCCTCAgcGTCTGGTTCAGGGCGCTGTGGAgcGTCAGG |
| *S87,92A* | TGAGgcTGAAGATGAAGAAgcCGAGGAAGAGTGAGAATTCAAGGCCTCTC | | CTCGgcTTCTTCATCTTCAgcCTCAACATCATCTTCATCACCTTGAATCA |
