# Supplementary material for: Phosphorylation of Def Regulates Nucleolar p53 Turnover and Cell Cycle Progression through Def Recruitment of Calpain3
Source: PLoS Biol. 2016 Sep 22;14(9):e1002555. doi: 10.1371/journal.pbio.1002555 (PMC5033581; doi:10.1371/journal.pbio.1002555)
Supplement: S7 Table — (DOCX) [file pbio.1002555.s021.docx]

| **S7 Table** | | |
| --- | --- | --- |
| **Construct** | **Forward primer (5’-3’) (Fw)** | **Reverse primer (5’-3’) (Rv)** |
| *S50A* | *as “S50A”* (Table S4) | *as “S50A”* (Table S4) |
| *S58A* | *as “S58A”* (Table S4) | *as “S58A”* (Table S4) |
| *S62A* | *as “S62A”* (Table S4) | *as “S62A”* (Table S4) |
| *S87A* | GATGATGTTGAGgcTGAAGATGAAGAAAGCGAGGAAGAGGATAACGAGGA | *as “S87A”* (Table S4) |
| *S92A* | GAAGATGAAGAAgcCGAGGAAGAGGATAACGAGGAGGAAGCTGAAGTTGA | TCCTCTTCCTCGgcTTCTTCATCTTCACTCTCAACATCATCTTCATCACC |
| *S58,62A* | *as “S58,62A”* (Table S4) | *as “S58,62A”* (Table S4) |
| *S87,92A* | TGAGgcTGAAGATGAAGAAgcCGAGGAAGAGGATAACGAGGAGGAAGCTG | *as “S87,92A”* (Table S4) |
| *S87,92E* | TGAGgaaGAAGATGAAGAAgaaGAGGAAGAGGATAACGAGGAGGAAGCTG | CTCttcTTCTTCATCTTCttcCTCAACATCATCTTCATCACCTTGAATCA |
