# Supplementary material for: Phosphorylation of Def Regulates Nucleolar p53 Turnover and Cell Cycle Progression through Def Recruitment of Calpain3
Source: PLoS Biol. 2016 Sep 22;14(9):e1002555. doi: 10.1371/journal.pbio.1002555 (PMC5033581; doi:10.1371/journal.pbio.1002555)
Supplement: S8 Table — (DOCX) [file pbio.1002555.s022.docx]

| **S8 Table** | | |
| --- | --- | --- |
| **Construct** | **Forward primer (5’-3’) (Fw)** | **Reverse primer (5’-3’) (Rv)** |
| *p53 ^A138V^* | TTTGCCAACTGGtCAAGACCTGCCCTGTGCAGCTGTGGGTTGATTCCACA | GGGCAGGTCTTGaCCAGTTGGCAAAACATCTTGTTGAGGGCAGGGGAGTA |
| *p53 ^R175H^* | AGGTTGTGAGGCaCTGCCCCCACCATGAGCGCTGCTCAGATAGCGATGGT | TGGTGGGGGCAGtGCCTCACAACCTCCGTCATGTGCTGTGACTGCTTGTA |
| *p53 ^M237I^* | CTACAACTACATcTGTAACAGTTCCTGCATGGGCGGCATGAACCGGAGGC | GGAACTGTTACAgATGTAGTTGTAGTGGATGGTGGTACAGTCAGAGCCA |
| *p53 ^R248W^* | GGCGGCATGAACtGGAGGCCCATCCTCACCATCATCACACTGGAAGACTC | GGATGGGCCTCCaGTTCATGCCGCCCATGCAGGAACTGTTACACATGTAG |
| *p53 ^R273P^* | GCTTTGAGGTGCcTGTTTGTGCCTGTCCTGGGAGAGACCGGCGCACAGAG | CAGGCACAAACAgGCACCTCAAAGCTGTTCCGTCCCAGTAGATTACCAC |
