# Supplementary material for: Phosphorylation of Def Regulates Nucleolar p53 Turnover and Cell Cycle Progression through Def Recruitment of Calpain3
Source: PLoS Biol. 2016 Sep 22;14(9):e1002555. doi: 10.1371/journal.pbio.1002555 (PMC5033581; doi:10.1371/journal.pbio.1002555)
Supplement: S9 Table — (DOCX) [file pbio.1002555.s023.docx]

| **S9 Table** | | |
| --- | --- | --- |
| **Construct** | **Forward primer (5’-3’) (Fw)** | **Reverse primer (5’-3’) (Rv)** |
| *CAPN3^C129S^* | AGCTAGGGGACtccTGGTTTCTCGCAGCCATTGCCTGCCTGACCCTG | GCGAGAAACCAggaGTCCCCTAGCTCTCCTTGACAGATGTCAGTTCTG |
| *CAPN3^C129S(-NOLS)^* | CGGCCAGTGAAACCCATCATCTTCGTTTCGGACAGAGCAAACAGCAAC | GAAGATGATGGGTTTCACTGGCCGATCCACGGAGATGGTATTTTCAAC |
| *CAPN3a^C110S^* | GATCTGGGTGACtccTGGTTGCTTGCAGCCATTGCCTGTCTGACTC | CTGCAAGCAACCAggaGTCACCCAGATCTCCCTGGCAGATATCAGTC |
| *CAPN3b^C120S^* | ACCTGGGAGACtctTGGCTGTTGGCTGCCATAGCTTGCCTGACCCTG | CCAACAGCCAagaGTCTCCCAGGTCTCCTTGGCAGATGTCAGTCC |
